# Bioavailable Nutrients (N and P) and Precipitation Patterns Drive Cyanobacterial Blooms in Missisquoi Bay, Lake Champlain

**DOI:** 10.3390/microorganisms9102097

**Published:** 2021-10-04

**Authors:** Sukriye Celikkol, Nathalie Fortin, Nicolas Tromas, Herinandrianina Andriananjamanantsoa, Charles W. Greer

**Affiliations:** 1 Department of Natural Resource Sciences, McGill University, Sainte-Anne-de-Bellevue, QC H9X 3V9, Canada; 2 Energy, Mining and Environment Research Centre, National Research Council Canada, Montreal, QC H4P 2R2, Canada; Nathalie.Fortin@cnrc-nrc.gc.ca; 3 Department of Biological Sciences, University of Montreal, Montreal, QC H2V 0B3, Canada; nicolas.tromas@umontreal.ca (N.T.); hn.andriananjamanantsoa@umontreal.ca (H.A.)

**Keywords:** bioavailable nutrients, nitrogen, phosphorus, precipitation, cyanobacterial blooms, *Dolichospermum*, *Microcystis*

## Abstract

Anthropogenic activities release large amounts of nitrogen (N) and phosphorus (P) nutrients into the environment. Sources of nutrients include surface and sub-surface runoffs from agricultural practices with the application of chemical fertilizers and manure as well as combined sewer overflows (CSOs). Nutrient runoffs contribute to the eutrophication of aquatic ecosystems and enhance the growth of cyanobacteria. Precipitation is an important driving force behind the runoff of nutrients from agricultural fields into surrounding water bodies. To understand the dynamics between nutrient input, precipitation and cyanobacterial growth in Missisquoi Bay, Lake Champlain (Quebec), one location in Pike River (a major tributary into the bay) and four locations in Missisquoi Bay were monitored from April to November in 2017 and 2018. Biweekly water samples were analyzed using chemical methods and high-throughput sequencing of 16S rRNA gene amplicons. High concentrations of N and P were typically measured in April and May. Three major spikes in nutrient concentrations were observed in early and mid-summer as well as early fall, all of which were associated with intense cumulative precipitation events of 40 to 100 mm within 7 days prior to sampling. Despite the high concentrations of nutrients in the spring and early summer, the cyanobacterial blooms appeared in mid to late summer as the water temperature increased. *Dolichospermum* sp. was the major bloom-forming cyanobacterium during both summers. A second intense bloom event of *Microcystis* was also observed in the fall (October and November) for both years. Variation in the cyanobacteria population was strongly associated with inorganic and readily available fractions of N and P such as nitrites and nitrates (NOx), ammonia (NH_3_) and dissolved organic phosphorus (DOP). During blooms, total Kjeldahl nitrogen (TKN) and total particulate phosphorus (TPP) fractions had a substantial influence on total nitrogen (TN) and total phosphorus (TP) concentrations, respectively. The abundance of bacteria involved in the metabolism of nitrogen compared to that of phosphorus revealed the importance of nitrogen on overall microbial dynamics as well as CB formation in the bay. Our findings emphasize the combined influence of precipitation events, temperature and several bioavailable fractions of nitrogen and phosphorus on cyanobacterial bloom episodes.

## 1. Introduction

Excessive growth of cyanobacteria in freshwater environments has been a worldwide concern for decades regardless of the climatic zone. The driving force behind cyanobacterial blooms (CBs) is the availability of nitrogen (N) and phosphorus (P) at elevated concentrations and at certain ratios [1]. Although the theory seems simple, delineating the source of nutrients, their routes of transportation and fate with respect to environmental (physical, chemical and biological) variables and how these interactions reflect cyanobacterial dynamics is complex.

Land use is a primary factor that determines the point and non-point sources of pollutants. Agricultural runoff of chemical and manure-based fertilizers, animal farming as well as combined sewer overflows (CSOs) are major contributors of nutrients into water bodies [2,3,4,5,6,7]. Modeling studies showed that land use could explain up to 74% of the nitrite-nitrate content in water [3]. The composition of the fertilizer (i.e., N- or P-based) determines the type of nutrient that will reach the water body [8]. Manure-based fertilizers are rich in both N and P and can harbour *E. coli* and other types of pathogens. The ratio of N and P is dependent on the animals from which the manure is obtained [9]. Wastewater is rich in organic P, soluble reactive phosphorus (SRP), organic N and NH_3_ [10]. In more recent years, persistent organic pollutants (POPs) such as herbicides, pesticides, polycyclic aromatic hydrocarbons (PAHs) and pharmaceutical and personal care products have been the focus of numerous research activities. These POPs are typically released in receiving waters via wastewater effluents, agricultural and industrial runoffs and have been shown to contribute to the growth of cyanobacteria [11,12,13,14,15] and the release of microcystin [16].

Precipitation events have a key role in introducing nutrients, especially nitrogenous components, to water bodies via agricultural runoff [17,18]. Fertilizer application on agricultural fields prior to rain is sometimes performed to ensure the penetration of nutrients into the soil. This strategy can be beneficial only when precipitation is light (in the form of drizzling) but highly detrimental during heavy precipitation and extreme events since it enhances the leaching of soluble nutrients, i.e., soluble N and P fractions, into surface waters [9]. Heavy precipitation also leads to stormwater runoff as well as overflows of domestic sewage and industrial wastewater, which contribute to the nutrient load in rivers and lakes. For example, the Bedford wastewater treatment plant, located in Pike River, recorded 271 overflows in 2017 and 243 in 2018 that were associated with precipitation [19].

The effects of rainfall patterns and climate change on cyanobacterial dynamics have been investigated by long-term field data and water quality models. The findings show that high frequency, intense rainfalls followed by long dry periods (4 to 10 days) are ideal for cyanobacterial growth [20]. However, frequent heavy rains also have the potential of flushing cyanobacteria and cause de-stratification. Conversely, a high number of small rainfall events could promote growth by continuously providing readily available nutrients [21,22,23]. While the impact of precipitation on the runoff of nutrients is well known and has been extensively modeled [24,25,26,27], the sequence of events from fertilizer application to precipitation and changes in N and P concentrations in water has not been well documented.

Lake Champlain has been studied in several aspects such as phosphorus bioavailability [28,29], nitrogen regeneration [30,31] and impacts of climate change [32]. Most of the previous research is based on environmental parameters such as nutrient input and atmospheric variables and overlooks the microbial diversity. Recent studies on Missisquoi Bay, Lake Champlain, employed advanced statistical analyses to investigate the occurrence and characteristics of cyanobacterial blooms [33] and niche separation [34] based on 16S rRNA gene amplicon sequencing, nutrient fractions, i.e., total, particulate and dissolved N and P, precipitation and temperature [33,34]. Redundancy analyses of the microbial diversity and environmental variables indicated that particulate nitrogen and particulate phosphorus were the most explanatory nutrient fractions related to the bloom [33]. Amplicon sequencing analysis revealed that *Polynucleobacter* C-subcluster had a reverse growth profile compared to *Dolichospermum* and *Microcystis* and could potentially indicate the onset of a bloom. Different bloom-forming cyanobacteria (*Dolichospermum* and *Microcystis*) had distinct nutrient preferences at the species and genus levels [34]. The niche separation of *Dolichospermum* was towards particulate N and P and precipitation, whereas the prevalence of *Microcystis*, an important microcystin producer in the bay was dependant on dissolved N and temperature [34]. This study demonstrated the association of particulate N and the incidence of cyanobacterial blooms. The strong correlation of blooms and particulate N and P is not surprising since they represent organic fractions of nutrients as well as biomass. 

A lot of effort and financial resources have been invested over the years to reduce the eutrophication of Lake Champlain with a focus mainly on P. To further control and reduce P loading from point and non-point sources and to meet the guidelines of the EPA Clean Water Act, an agreement between Quebec and the state of Vermont, USA was renewed in April 2021 to reduce the annual in-lake total P (TP) concentrations in Missisquoi Bay to 25 μg/L [19]. TP and total N (TN) consist of both the organic and particulate fractions of P and N that are difficult to breakdown and metabolize, as well as the inorganic and soluble (dissolved) fractions that are readily available to microorganisms. It is important to note that the major components of bacterial cells are organic N and P. TN and TP values are, therefore, highly influenced by the organic and particulate N and P concentrations that are related to cellular biomass, especially during CBs. While the TN:TP ratio is a universal indicator of trophic status of aquatic ecosystems, it overlooks the various nutrient fractions and could be biased during intense CB events. For a deeper insight into the nutrient-bloom dynamics, it is ideal to evaluate, in addition to TN and TP, the soluble and readily bioavailable fractions of N and P as well as the organic and particulate components.

**Figure 1:**
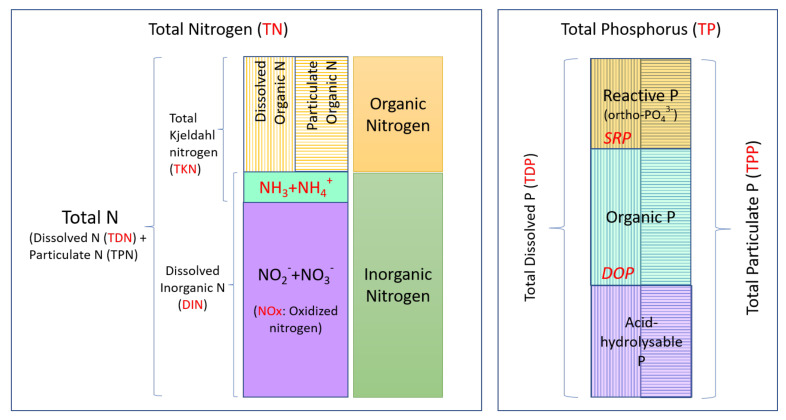
Nitrogen and phosphorus fractions in water. TN and TP fractions are mainly classified as dissolved and particulate and further classified as organic and inorganic. Vertical lines represent the dissolved fractions and horizontal lines represent the particulate fractions of N and P. The fractions monitored in this study are indicated in red.

Fractionations of N and P are depicted in Figure 1. In the context of this study, the nitrogen fractions that were monitored included ammonia (NH_3_), oxidized nitrogen (NOx; total of nitrites (NO_2_^-^) and nitrates (NO_3_^-^)), dissolved organic N (DON), total dissolved N (TDN), total Kjeldahl nitrogen (TKN) and total N (TN). The phosphorus fractions monitored in this study were soluble reactive phosphorus (SRP), dissolved organic P (DOP), total dissolved P (TDP), as well as total particulate P (TPP) and total P (TP).

The latest studies on Missisquoi Bay, Lake Champlain, offer valuable insights about biotic factors and environmental variables (nutrients, temperature and precipitation) for the prediction and occurrence of cyanobacterial blooms [33,34]. This study fills the knowledge gap in previous findings by (i) exploring various fractions of bioavailable and particulate nitrogen and phosphorus; (ii) tracking a wider selection of environmental variables such as depth, pH and chlorophyll-a; (iii) monitoring additional sampling sites (a river, river mouth and the shore) to understand the fate of nutrients and their impact on the intensity of blooms. We hypothesized that the bioavailable nutrients, both N and P, are the key environmental components that lead to cyanobacterial blooms and that precipitation has a significant role in their transportation to the lake.

## 2. Materials and Methods

### 2.1. Sampling

**Figure 2:**
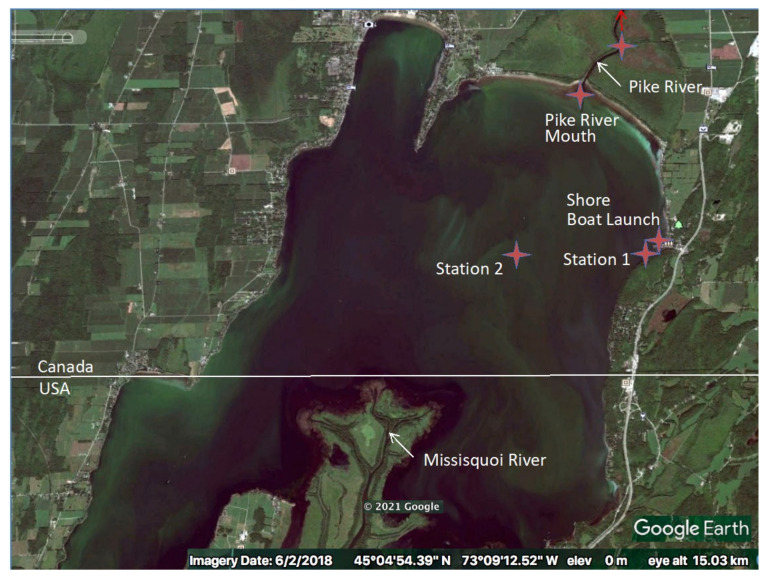
Sampling locations in Missisquoi Bay, Lake Champlain, in 2017 and 2018. Google Earth, 2 June 2018 [35]. Stations include the tributary Pike River (PR); Pike River mouth (PRM); Littoral Station 1 (St1); Pelagic Station 2 (St2) and Shore Boat launch (SBL).

Water samples were taken on a biweekly basis between April and November in 2017 and 2018, from four locations (Pike River mouth [PRM], Littoral Station 1 [St1], Pelagic Station 2 [St2], Shore Boat launch [SBL]) in Missisquoi Bay, Lake Champlain, Quebec, and one location in Pike River (PR), a tributary flowing into the northern part of Missisquoi Bay. St1 was located above the water intake of a drinking water treatment plant. A map of the sampling locations is presented in Figure 2 [35].

PR samples, SBL samples and St1 April, May and October samples were taken from the surface water. The photic zone for St1, St2 and PRM was calculated based on Secchi disk measurements. Composite samples from four different depths within the photic zone were combined in a 10L bucket using a Rule IL280P Slimline Submersible and Inline pump connected to Teflon tubing and a battery. The composite samples were well mixed prior to filling each sterile glass bottle. The bottles were kept at 4 °C during transport. The total number of samples taken from each sampling site is presented in Table 1.

### 2.2. In Situ Data Collection and Precipitation

Physicochemical water quality parameters (temperature, dissolved oxygen, pH, conductivity, chlorophyll-a, phycocyanin) were recorded each 50 cm of depth using a YSI 6600 v2 probe (Hoskin Scientific, Oakville, ON, Canada). Solar and UV irradiance were measured using Apogee Instruments MP-200 and MU-200, respectively (Hoskin Scientific, Oakville, ON, Canada). Atmospheric variables (temperature, wind, humidity, atmospheric pressure) were measured using a Kestrel 3500 weather meter (Nielsen-Kellerman, Bothwyn, PA, USA).

Cumulative precipitation in the vicinity of Missisquoi Bay, 1 to 7 days prior to our sampling campaigns was calculated with rain data retrieved from Farmzone [36] and the Weather Network [37] (Table S1). Rainfall categories used in this study were based on our previous work [2].

### 2.3. Nutrient Analyses

Duplicate samples were taken for nutrient analyses. Thoroughly mixed water samples were transferred into HDPE bottles for nitrogen and phosphorus analyses and into 40 ml borosilicate glass vials for DOC and TOC. DOC, NO_2_^−^, NO_3_^−^, NH_4_ and SRP samples were filtered on site through pre-hydrated 0.45 µM Filtropur S, PES syringe filters (Sarstedt, Montreal, QC, Canada). These nutrient samples were stored at 4 °C during transport. Every sample was stored and processed within the time frame recommended in the APHA Standard Methods [38]. The concentrations of TN, TDN, nitrate and nitrite (method EPA353.2) and the NH_3_ (method EPA350.1) were measured on a Lachat, Quickchem 8500. The TP, TDP and SRP (Method EPA365.3) were measured on an Astoria-Pacific, Astoria 2. The DOC (method EPA415.1) was measured on an OI Instrument, Aurora 1030. The chlorophyll-a (extraction with 95% ethanol and absorbance measurement at 665 nm) was measured on a Spectronic, Genesys 10 spectrophotometer. Total Kjeldahl nitrogen (TKN) was calculated by subtracting nitrite and nitrate concentrations from TN. Dissolved inorganic nitrogen (DIN) was calculated by summing nitrite, nitrate and ammonia concentrations. TPP was calculated by subtracting TDP from TP.

### 2.4. DNA Extraction

Water samples were mixed thoroughly prior to each filtration. Triplicate water samples of 130 to 250 mL, depending on the planktonic biomass, were filtered through 0.2 μm hydrophilic polyethersulfone membranes (Millipore, Etobicoke, ON, Canada). The filters were stored at −80 °C until further analysis. The DNeasy Power Water Kit was used for DNA extractions (Qiagen, Toronto, ON, Canada). The manufacturer’s protocol was followed with slight modifications. The filters were incubated at 65 °C for 10 min to facilitate the lysis of cyanobacterial cells. For each sample, the DNA pellet was resuspended in 100 μL of TE (Tris-Cl, 10 mM; EDTA, 1 mM; pH 8) and stored at −20 °C. To eliminate the RNA, 50 μL of DNA was treated with RNase If (New England Biolabs, Whitby, ON, Canada) according to the manufacturer’s protocol. The purified DNA was quantified using the PicoGreen^®^ dsDNA quantitation assay (Invitrogen, Burlington, ON, Canada) and a Safire microplate detection system (Tecan, Männedorf, Switzerland). DNA was normalized to 1 ng/μL prior to 16S rRNA gene library preparation.

### 2.5. Taxonomy

Water samples (20 mL) were transferred into brown borosilicate vials and preserved with 1 mL Lugol’s iodine solution at 4 °C until analysis. Preserved samples were analyzed according to APHA Method 10200F [39]. A preliminary scan of a 1 mL aliquot in a Sedgewick-Rafter (S-R) counting chamber was performed to assess cyanobacterial concentration. If few cyanobacteria were observed in the S-R chamber, a 5, 10 or 25 mL aliquot was settled in an Utermohl counting chamber. After the appropriate settling time, at least 30 random fields were counted from each Utermohl chamber (field number was adjusted to reach a target of 300 cells, filaments or colonies in a reasonable amount of time). If cyanobacterial density was very high, counts were performed using a Sedgewick-Rafter counting chamber. At least 30 random fields or 4 strips were examined, with a target of 300–400 natural units (cells, colonies or filaments). Only “live” cells (those containing protoplasm) were counted. Cyanobacteria were counted using an Olympus inverted microscope at 500× magnification. Species identification was aided by examination with an oil immersion objective on a light microscope at 1000×. Field area was delimited by a Whipple grid calibrated to the objective lens. Density was reported as cells/ml. Identifications were made using standard bench references [40,41,42]. The number of cells in colonial and filamentous forms was recorded. Biovolume (µm^3^/cell) was estimated by measuring the linear dimensions of up to 20 individuals per species and calculating the average volume based on standard geometric formulas [43].

### 2.6. 16S rRNA Gene Library Preparation and Sequencing

DNA libraries for paired-end Illumina sequencing were prepared using a two-step 16S rRNA gene amplicon PCR as described in Preheim et al. [44]. We amplified 292 bp of the 16S rRNA gene V4 region (one replicate) using 2 ng of DNA and the U515_forward (5’ ACAC GACG CTCT TCCG ATCT YRYR GTGC CAGC MGCC GCGG TAA 3’) and E786_reverse primers (5’ CGGC ATTC CTGC TGAA CCGC TCTT CCGA TCTG GACT ACHV GGGT WTCT AAT 3’) and then confirmed the library size by agarose gels [33]. DNA quantification of the libraries was performed with a Qubit v.2.0 fluorometer (Life Technologies, Burlington, ON, Canada). Libraries were pooled and denatured as described in the Illumina’s 16S Metagenomic Sequencing Library Preparation Guide (Part# 15044223 Rev. B). We performed two sequencing runs using MiSeq reagent Kit V2 (Illumina, San Diego, CA, USA) on a MiSeq instrument (Illumina). Each run included negative controls and mock communities (ATCC MSA-1002), which enabled us to optimize the sequencing workflow, providing reliable comparative data while improving assay consistency.

### 2.7. Sequence Analysis

The raw paired-end FASTQ reads were demultiplexed using idemp (https://github.com/yhwu/idemp/blob/master/idemp.cp; accessed on 20 June 2020). Illumina MiSeq reads were filtered using BBduk (http://bbtools.jgi.doe.gov, version 38.33; accessed on 20 June 2020) to remove Illumina adapters and known Illumina artifacts. We performed an aggressive trimming using the total length of the kmers for the search (k = 19), allowing only one mismatch (hdist = 1). We kept only reads with lengths greater than 220 base pairs (minlen = 220) and quality scores greater than 20 (maq = 20), retaining approximately 85% of total reads for each sample. After removing adapters, DADA2 was used to quality filter, trim, de-noise and merge the data [45]. All data sets were pre-processed separately by run (96 samples per run). Reads containing Ns (maxN = 0), shorter than 200 bp (minLen = 200) or greater than 240 bp (maxLen = 240) were discarded. All reads that matched against the phiX genome were discarded (rm.phix = TRUE). Error models were randomly calculated (randomize = TRUE) using 1e09 bases for each data set (nbases = 1e09). We obtained 12,466 amplicon sequence variants (ASVs) (8,452 ASVs for 2017 and 7,337 ASVs for 2018) from the 5,801,357 sequences processed through DADA2, ranging from 206 to 630,826 reads per sample, with a median of 222,299. Prior to the analysis, four samples with less than 1000 sequences were removed from the ASV table using Phyloseq yielding a final data set of 99 samples (R package version 1.30.0) [46]. Taxonomy was assigned using a combination of GreenGenes (version 13.8) and a freshwater- specific database (Freshwater database, 15 June 2020 release) [47], with TaxAss method [48]. We removed ASVs that were not prokaryotes but still present in the database (Cryptophyta, Streptophyta, Chlorophyta and Stramenopiles orders) yielding to a final of 10,952 ASVs.

To evaluate the accuracy of our analysis inferred with the DADA2 pipeline, we checked the output sequences from the “mock community” if they matched the reference sequences (ATCC MSA-1002). We were able to recover ~92% of the mock sequences for each run with low false positive rate (on average < 2%).

### 2.8. Microbial Diversity

We estimated Shannon diversity and total richness with DivNet and Breakaway, respectively, which account for sampling variation (DivNet R package version 0.3.6 [49]; Breakaway version 4.7.3 [50]). Statistical analyses were performed using betta to compare diversity between bloom and non-bloom samples [51].

### 2.9. Spatio-Temporal Analysis

The beta diversity was calculated using a non-rarefied ASV table and Jensen–Shannon divergence (JSD), a metric that is robust to sequencing depth variation [44,52]. We used the distance function from phyloseq R package (version 1.19.1) [46] and calculated the square root of each metric (JSD). Differences in community structure between groups was tested using permutational multivariate analysis of variance (PERMANOVA) [53] with the adonis function. As PERMANOVA tests can be sensitive to dispersion, we also tested for dispersion in the data by performing an analysis of multivariate homogeneity (PERMDISP) [54] with the permuted betadisper function. PERMANOVA and PERMDISP were performed using the R *vegan* package, version 2.4-1 [55], with 999 permutations.

### 2.10. ASVs Relationships with Environmental Parameters

We investigated how the environment could explain microbial community variation. We first analyzed how the environmental variables (Table S1) were correlated with one another using a Spearman correlation analysis. (*Hmisc* R package, version 4-5.0). P-values were corrected using p.adjust function and Holm procedure (*stats* R package). We then performed a redundancy analysis (RDA) [56] that searches for the linear combination of explanatory variables (environmental data) that best explains the variation in a response matrix (ASV table). The ASV table was transformed by Hellinger transformation [57] as advised in Legendre and Legendre [58]. The environmental matrix was z-score standardised using the function decostand (x, method = ’standardise’) because different environmental parameters are in different units. To select the significant explanatory variables, we performed a forward selection using the ordiR2step function (*vegan* R package). We again ran an RDA but only with the significant variables. The function vif.cca (*vegan* R package) was used to estimate the variance inflation factors and assess co-linearity among the selected explanatory variables. To determine the significance of constraints, we used the anova.cca function from the R *vegan* package and calculated the adjusted R2 of the RDA using the RsquareAdj function (*vegan* package).

### 2.11. Cyanobacterial Response to Environmental Variables

Following the method described in Tromas et al. [34], we analyzed the response of the most dominant cyanobacterial genera to environmental variables. We used a Latent Variable Model (LVM) framework (boral package in R,) on *Dolichospermum* and *Microcystis* abundances that were, for this analysis, centered log ratios transformed to correct for data compositionality [59,60].

## 3. Results

### 3.1. Nutrient Concentrations, Precipitation and Cyanobacterial Blooms

Nitrogen fractions (DON, NH_3_, TKN, NOx, TN, TDN) and phosphorus fractions (SRP, DOP, TDP, TPP, TP) were monitored for the samples taken from April to November in 2017 and 2018. N and P fractionation is depicted in Figure 1 and ranges of N and P are given in Tables S2 and S3. Due to yearly variations in N and P profiles, the data were analyzed and presented separately for the 2017 and 2018 sample sets.

**Figure 3:**
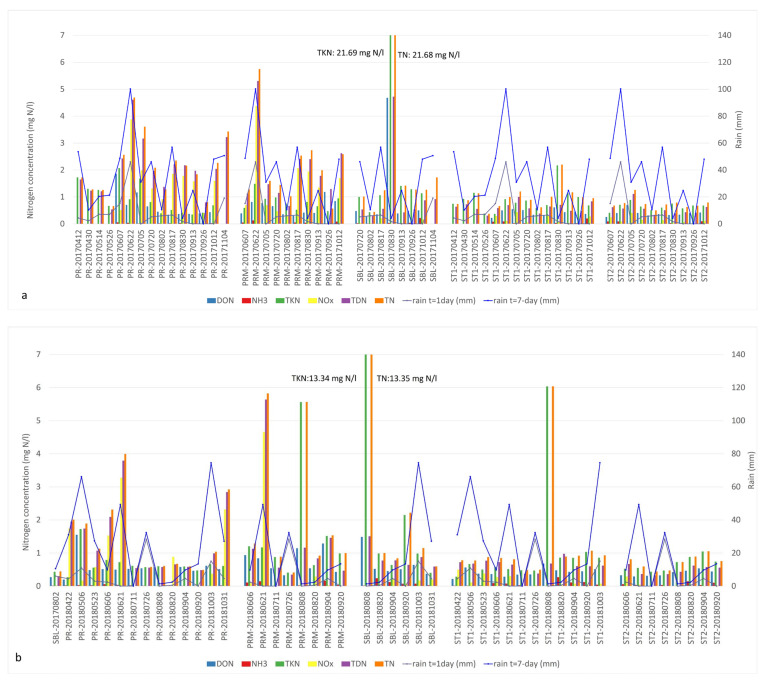
Nitrogen fractions and cumulative precipitation identified in Missisquoi Bay during (**a**) 2017 and (**b**) 2018 sampling campaigns. Occasional spikes of NOx and TDN concentrations in PR and PRM sites in June and October in both years corresponded to high cumulative rain. Extreme TKN values corresponded to intense bloom episodes. Concentrations exceeding the value axis are indicated in the text boxes.

**Figure 4:**
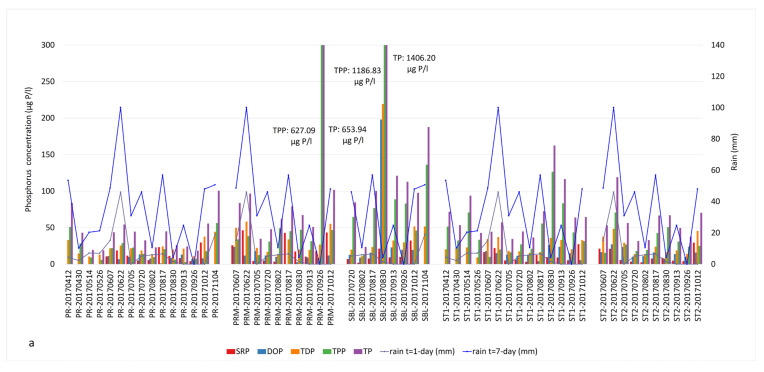

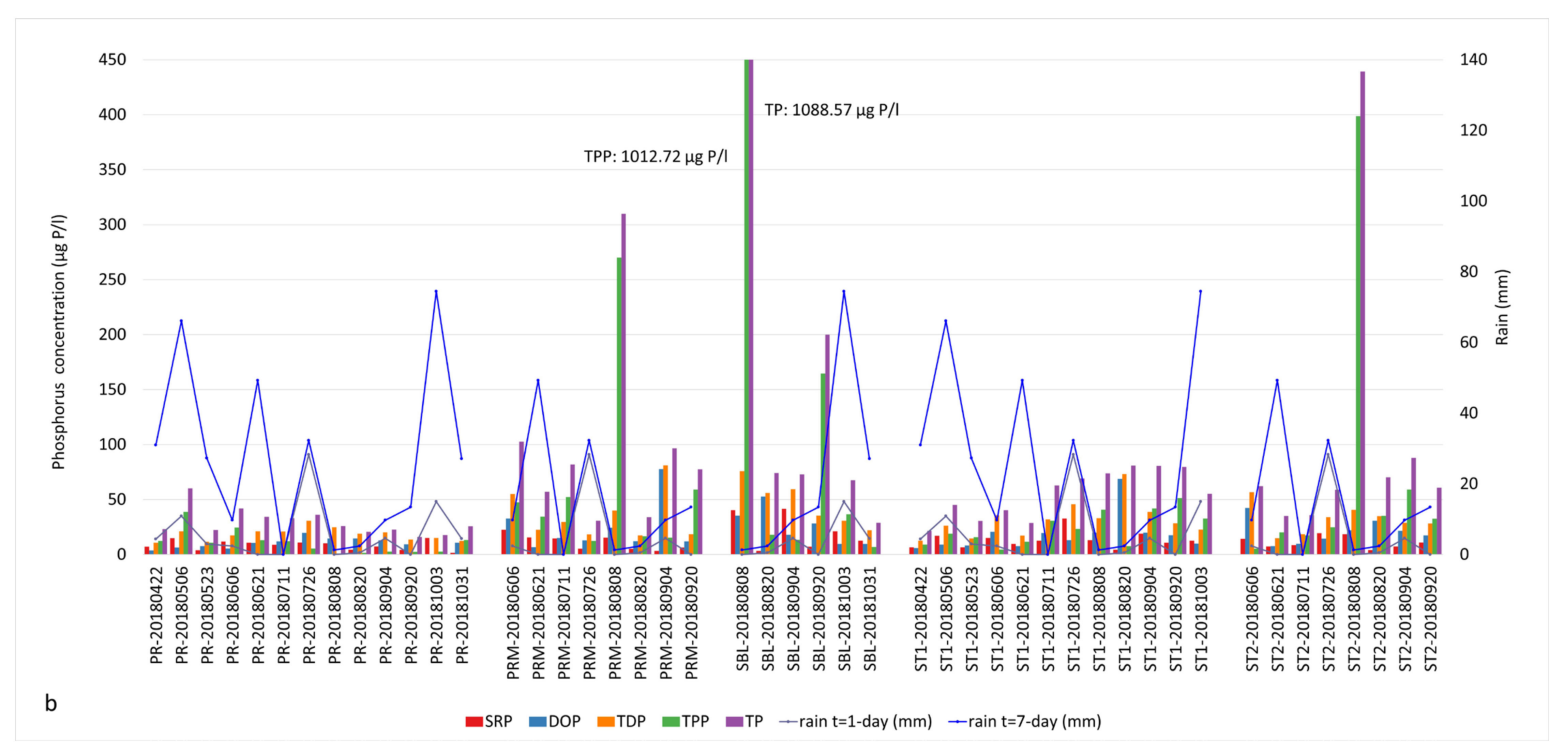
Phosphorus fractions and cumulative precipitation identified in Missisquoi Bay during (**a**) 2017 and (**b**) 2018 sampling campaigns. Extreme TPP values corresponded to intense bloom episodes. Concentrations exceeding the value axis are indicated in the text boxes.

Nutrient profiles per sample with respect to cumulative precipitation (t = 1 day and t = 7 days prior to sampling) were investigated to understand the impact of precipitation on nutrient flux in the river and the bay (Figure 3 and Figure 4). Correlations between the CB periods and N:P ratios of various nutrient fractions were also explored (Table S4).

The 2017 sampling season was associated with multiple rain events (Figure 3a). Important rainfall events with 7-day accumulation of more than 40 mm were observed every month with the exception of May and September. In PR, the abundant N fractions observed in early spring included DON, TDN and TKN while NOx levels were low (0 to 0.5 mg N/L). The highest concentrations of DON and TKN in the river were 1.86 and 2.07 mg N/L, respectively, at the beginning of June (Tables S1 and S2). Their concentrations were very similar, which indicates that the dominant forms of N were organic. High concentrations of NOx (3.87 mg N/L) were measured on June 22 in both PR and PRM following significant precipitation that contributed to a 7-day rain accumulation of 100 mm (Tables S1 and S5). In the 2017 samples, the dominant fractions of bioavailable N identified in PR and PRM throughout the sampling season were NOx and TDN, whereas TKN was the principal form of N in St1, St2 and SBL samples (Figure 3a). The highest concentrations of various forms of nitrogen that year were measured in the SBL station at the end of August during a bloom event. On that day, DON, TKN and TN concentrations were 4.68 and 21.70 and 21.68 mg/l, respectively (Tables S1 and S2). These concentrations showed that TKN was the major nitrogen fraction and consisted of particulate organic nitrogen.

In 2018, substantial rainfall events were observed in May, June and October. The inputs of nitrogen observed in the river were also associated with significant rainfall events prior to our sampling campaigns (Figure 3b). Peaks of NOx concentrations in the river (PR) were measured in May (1.73 mg N/L) and October (2.32 mg N/L). Similarly, important NOx inputs were measured on June 21, in both PR and PRM with 3.27 and 4.65 mg N/L, respectively (Tables S1 and S2). The major available N fractions in the PR tributary included DON, TKN, NOx and TDN throughout our sampling season. Very high concentrations of TKN, TN and TDN were also measured on multiple occasions in all of our stations with the exception of the pelagic station 2 (Figure 3b, Table S1).

In both 2017 and 2018, the cumulative rain profiles followed a parallel trend with the dissolved nitrogen fractions in PR and PRM sites compared to St1, St2 and SBL (Figure 3a,b, Table S1). Spikes of NOx and TDN were observed in PR along with peaks between 49.3 and 100.1 mm of 7-day cumulative rain, specifically at the beginning of the summer (second half of June). For example, the concentrations of TDN for PR during that sampling event were 4.61 and 3.79 mg N/L in 2017 and 2018, respectively. In PRM, the concentrations of TDN were 5.30 mg N/L in 2017 and 5.64 mg N/L in 2018 (Table S1). The same trend was observed for both years, later in the fall with cumulative rain between 48 and 74.5 mm in October. Concentrations between 2.0 and 2.8 mg N/L were identified in PR and concentrations of 2.6 mg N/L were measured in PRM on 12 October 2017 (Table S1).

In this study, the bloom status was determined by microscopic counts of cyanobacteria and represented samples with a total biovolume of >20,000 µm^3^/mL. Cyanobacterial blooms were observed in 41 out of our 104 samples (Table S1).

During both years, the PRM, St1, St2 and SBL stations were associated with the presence of bioavailable DON and TDN fractions in the early summer, and later by TKN as the growth of bacterial biomass increased (Figure 3). Cyanobacterial bloom events were associated to high TKN-TN concentrations and low precipitation. The DON and TDN concentrations were very similar during bloom events, indicating that organic nitrogen was the major form of dissolved nitrogen. The highest TKN concentrations correspond to the occurrence of intense CBs. The SBL station is an extreme example of this correlation with peak TKN concentrations of 21.7 mg N/L observed on 30 August 2017 and of 13.3 mg N/L on 8 August 2018. TKN and TN concentrations were equal on these dates (Figure 3, Table S1). 

The TP values measured in our 2017 and 2018 samples were significantly above the 25 µg/L target that is part of the agreement between Quebec and the state of Vermont concerning phosphorus reduction in Missisquoi Bay [19]. Phosphorus fractions and cumulative rain profiles are presented in Figure 4 and Tables S1 and S3.

In 2017, the major forms of bioavailable P were TDP and SRP in the PR tributary during our June and October sampling campaigns following significant precipitation. The concentrations of TDP were 25.5 and 37.7 µg/L on June 21 and October 12 (Table S1). The SRP concentrations reached 18.7 and 29.6 µg/L on those dates (Figure 4a, Table S1). The SBL station showed critical concentrations of TPP (1186.83 µg/L) as well as TDP (219.37 µg/L) and DOP (198.12 µg/L), especially at the end of August (Figure 4a, Tables S1 and S3). These concentrations coincided with an intense bloom episode. Other samples also had important concentrations of TPP throughout the sampling season, associated with the occurrence of CBs. The SRP and TDP concentrations showed similar profiles to NOx in June, mid-August (before the bloom) and October (bloom lysis).

In 2018, both the river and the bay samples contained high concentrations of dissolved forms of P such as SRP (15.15 to 41.52 µg/L), DOP (19.73 to 77.71 µg/L) and TDP (30.69 to 81.05 µg/L) in early June and late July to mid-August (Figure 4b, Tables S1 and S3). The mid-August sampling campaign corresponded to the lysis of the first intense bloom of 2018. The impact of heavy precipitation on P runoff was observed on TDP and SRP fractions rather than TP (Figure 4b). Similar to 2017, TPP was the most dominant P fraction during the bloom episodes, indicating the presence of high cyanobacterial biomass.

In 2018, the trends of SRP and TDP compared to NOx were not as specific as 2017. Nevertheless, SRP concentrations were generally high during bloom periods, indicating a continuous bioavailability of P.

Finally, P concentrations were higher in PRM, St1, St2 and SBL than PR in both years (Figure 4, Table S1), most likely due to the release of P from the sediments.

We explored the ratios between various N and P components, focusing on the TN:TP ratio as a common indicator and the DIN:SRP ratio as the readily bioavailable nutrients (Table S4). The threshold value for the TN:TP ratio is typically 22 in freshwater, above which is P-limiting and below which is N-limiting [61]. In this study, 36 out of 41 bloom samples had a TN:TP ratio between 22 and 181, and 5 samples had TN:TP ratios between 2 and 20. The DIN:SRP ratios of the first intense blooms observed in August were in the range of 0.5–22 in 2017 and 2018, except for PRM in 30 August 2017, where the DIN:SRP ratio was 252. High DIN:SRP and TN:TP values corresponded to samples with high spikes of NOx concentrations in the water column or excessive TKN amounts due to a bloom event. In September 2017, a manure or septic waste spill was reported in the vicinity of the PRM station. This was reflected as extreme TP and TPP concentrations and the lowest TN:TP ratio of 2. On 26 September, the concentrations of TP and TPP in PRM water samples were 653.94 ug/L and 627.09 ug/L, respectively (Table S1 and Figure 4a).

### 3.2. Correlation of Environmental Variables

**Figure 5:**
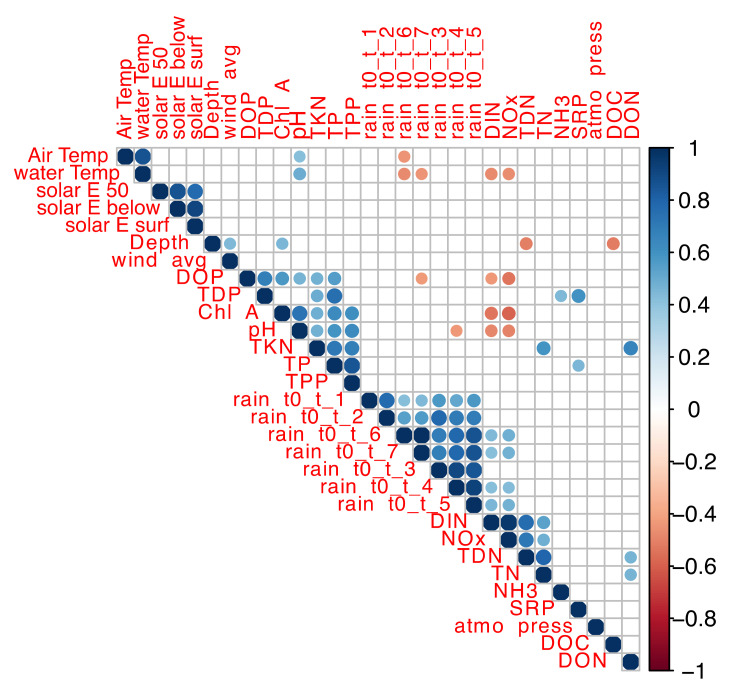
Correlation of environmental variables from 2017 and 2018 sampling campaigns. The color scale indicates the strength of correlation between environmental parameters. Blue scale indicates positive correlations; red scale indicates negative correlations.

Correlation of environmental variables with one another was determined using Spearman correlation analysis. Considering the yearly differences in N and P profiles, the analysis was first conducted in 2017 and 2018 data sets separately (Figures S1 and S2) to obtain a deeper insight into the bacterial dynamics for each year. Analyses of combined datasets (Figure 5) showed a positive correlation between cumulative rain and NOx and DIN and a negative correlation with DOP. Positive correlations between chlorophyll-a, pH, TKN, TPP and TP, strong indicators of CBs, were also identified. 

NOx and DIN were negatively correlated with water temperature indicating that DIN and NOx inputs were higher during the spring, as observed in the profile of nutrient concentrations (Figure 3, Table S1). In 2017, there was a stronger positive correlation between NH_3_, TDN, TN and SRP and a stronger negative correlation between DIN-NOx and pH-chlorophyll-a (Figure S1).

In 2018, air temperature, water temperature and DOP were negatively correlated with cumulative rain (Figure S2). The first intense blooms of 2017 and 2018 occurred when the 7-day cumulative rainfall was as low as 4.1 mm and 1.3 mm, respectively. This correlation and observation corroborate the model predictions that CBs occur during prolonged drought periods accompanied by high temperatures [21,22,62,63].

### 3.3. Microbial Community Analysis using 16S rRNA Gene Libraries

**Figure 6:**
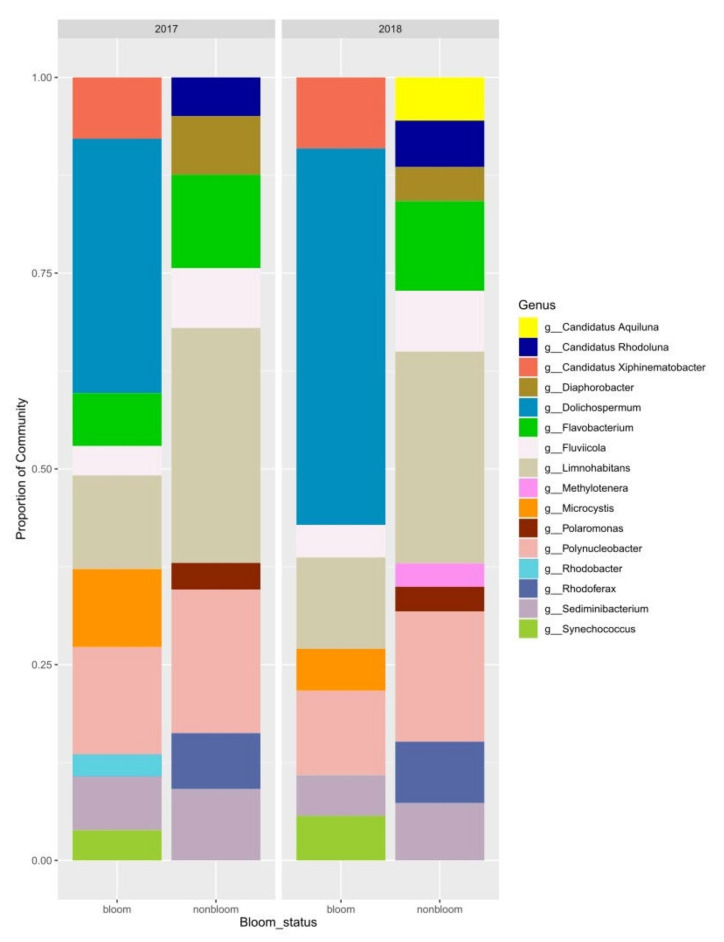
Dominant prokaryotic genera in bloom and non-bloom samples.

A total of 104 samples (57 samples from 2017, 47 samples from 2018) were sequenced, and 99 of them were analyzed after removing the samples with <1000 reads. A total of 12,466 ASVs were identified within the sample set (8,452 ASVs for 2017 and 7,337 ASVs for 2018). The most dominant phyla in the tributary river (PR) and lake samples (PRM, St1, St2, SBL) were Proteobacteria (38%), Actinobacteria (26%), Bacteroidetes (16%) and Verrucomicrobia (6%). The cyanobacteria (9%) were observed only in the lake samples (Figure 6). The most dominant genera in bloom and non-bloom samples for 2017 and 2018 are compared in Figure 6.

The major bloom forming cyanobacteria were *Dolichospermum* and *Microcystis*. *Synechococcus* co-occurred with *Dolichospermum* and *Microcystis* in some of the bloom samples. Distribution of the cyanobacteria per sampling site and strain-level identification by microscopy are presented in Figure S3. *Polynucleobacter, Limnohabitans, Sediminibacterium* and *Flavobacterium* were the most abundant genera in non-bloom samples. They were also present in high abundances during bloom events, suggesting a mutual relationship. *Polynucleobacter* sp. was present in up to 20% of non-bloom samples and in 10–12% of bloom samples. *Limnohabitans* sp. represented approximately 50% of the diversity in non-bloom samples for both years and represented 10% of the population in bloom samples. *Polaromonas* sp. had approximately 4% abundance in our April and May samples. *Rhodoferax sp* (7%) was present in non-bloom samples, particularly PR and St1 (Figure S4) in early spring for both years and in the river at the beginning of November 2017. *Candidatus Xiphinematobacter* represented 7 and 10% of the population in 2017 and 2018, respectively (Figure 6). *Flavobacterium* (12%) and *Fluviicola* (8%) were more abundant in non-bloom samples compared to bloom periods with 0 to 7% and 3 to 4% relative abundance, respectively (Figure 6).

**Figure 7:**
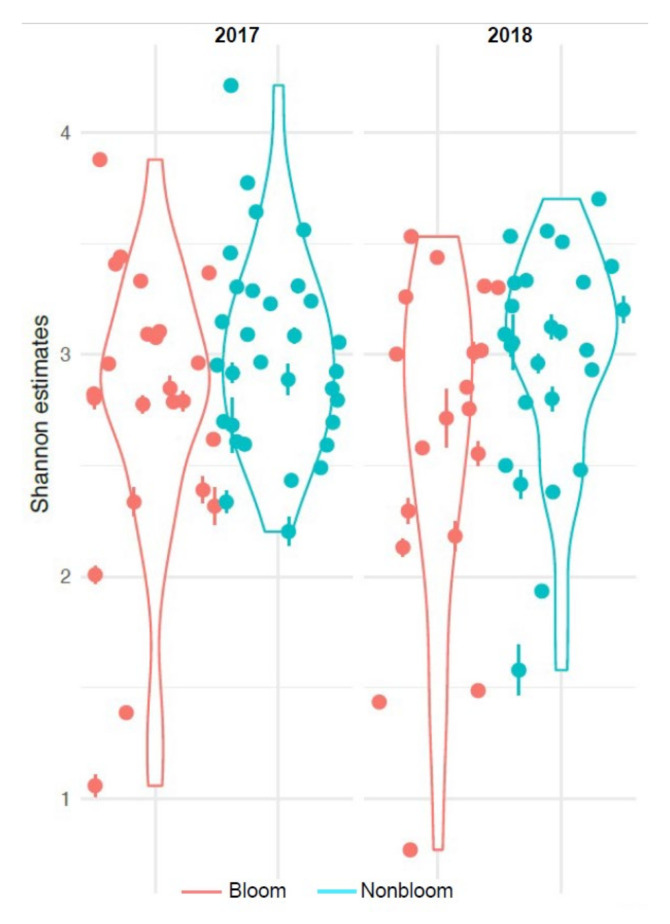
Shannon diversity index of 2017 and 2018 sample sets.

We compared the diversity between bloom and non-bloom samples in terms of species evenness and richness and observed a significant decrease in the Shannon diversity during blooms in both 2017 and 2018 (betta test, *p* < 0.001) (Figure 7). This decrease is likely directly associated with the bloom and a decrease in nutrients and oxygen in the surrounding water column, as was observed in previous studies [33] from the same sampling sites. As total richness was not different between these two groups (betta test, *p* = 0.11, Figure S5), the results suggest that evenness was impacted during blooms. 

### 3.4. Spatio-Temporal Analysis of Bacterial Community

**Figure 8:**
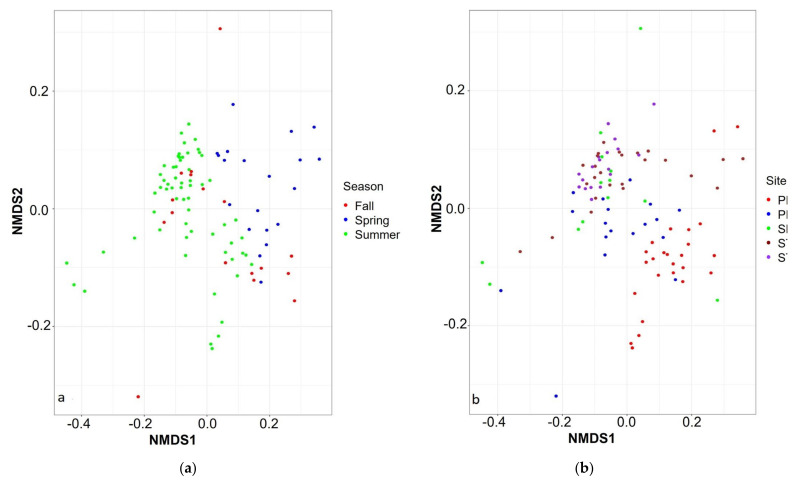
Jensen–Shannon divergence based on (**a**) seasons and (**b**) sampling sites.

The beta diversity was calculated using a non-rarefied ASVs table to determine Jensen–Shannon divergence (JSD) [44,52]. Differences in community structure between groups were tested using permutational multivariate analysis of variance with PERMANOVA. The data were also tested for dispersion by performing an analysis of multivariate homogeneity [54]. The results are presented in Table 2 and Figure 8.

The microbial diversity was clustered based on temporal and spatial variables (Table 2). Week (R^2^ = 0.425) and day of the year (R^2^ = 0.424) were the best explanatory temporal variables, whereas there was no significant difference between years (R^2^ = 0.017). Fall and summer samples clustered together compared to spring samples where CBs did not occur (Figure 8a). Sampling site was the most significant spatial variable (R^2^ = 0.167,) compared to depth (R^2^ = 0.109). The St1, St2 and SBL samples were clustered together compared to PR (Figure 8b). The diversity of PRM, a transition zone between PR and the bay area (St1, St2 and SBL), clustered with PR during the non-bloom period (spring) and with the bay area during the blooms (summer and fall) (Figure 8a).

### 3.5. Bacterial Community and Environmental Relationships

**Figure 9:**
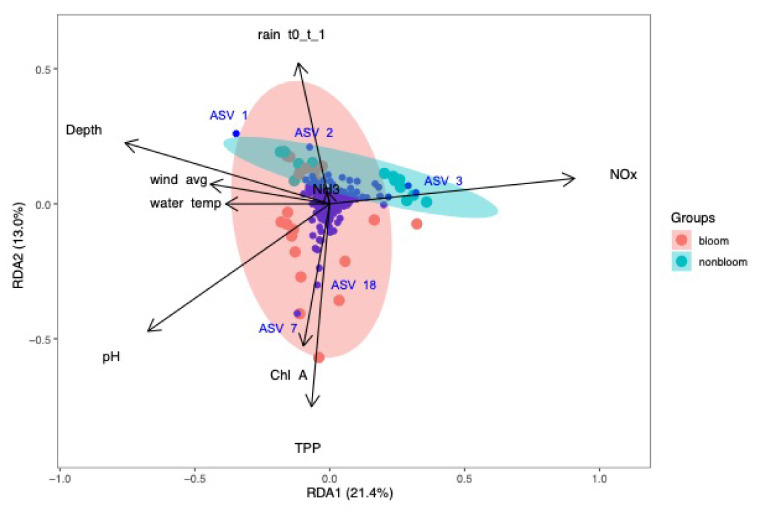
Redundancy analysis of bacterial community and environmental parameters for samples collected in 2017. NOx had a strong influence on non-bloom community. TPP, chlorophyll-a and pH were associated with the bloom samples. NH_3_ and rain were positively correlated.

**Figure 10:**
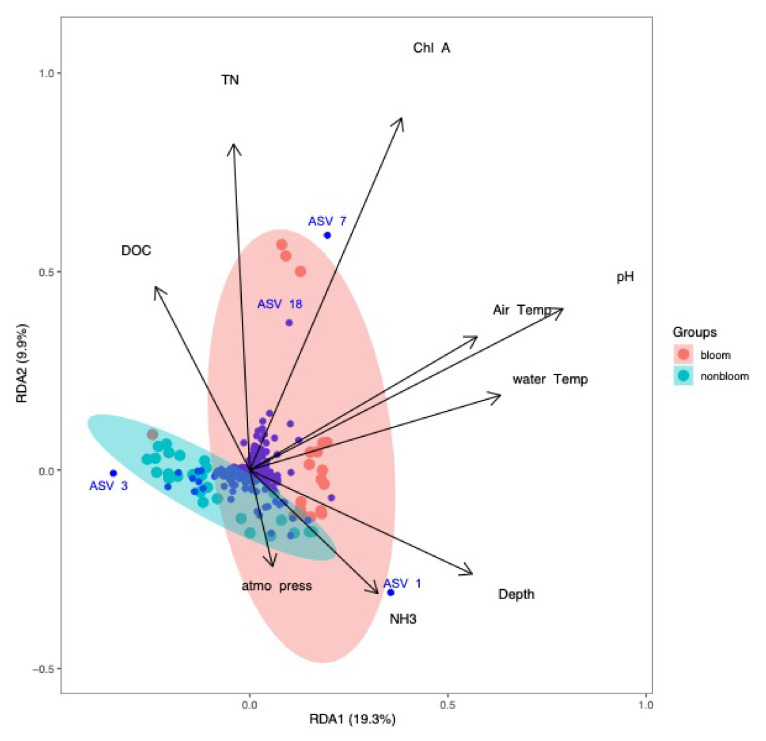
Redundancy analysis of bacterial community and environmental parameters for samples collected in 2018. NH_3_ had a positive correlation with the non-bloom bacterial community. The bloom samples were strongly associated with TN, DOC, chlorophyll-a, pH and temperature.

The relationships between the bacterial community (ASVs) and environmental parameters were investigated via redundancy analysis (RDA, Table S6) [56]. The 2017 and 2018 sample sets were assessed separately for a detailed understanding of the annual variations and are presented in Figure 9 and Figure 10. The results showed that the microbial diversity was influenced by different environmental parameters each year.

In 2017, RDA plots showed that the most significant environmental parameters could explain 34.4% (RDA1:21.4%, RDA2: 13.0%, adjusted R^2^=40.9%) of the variations in microbial community (Figure 9). ASV_1 and ASV_2 belonged to the ACK-M1 family of Actinomycetales, and ASV_3 corresponded to *Limnohabitans* sp. All were associated with the non-bloom community and strongly influenced by NOx, NH_3_ and depth. Wind speed, depth and water temperature were negatively correlated to NOx. These findings are in agreement with the Spearman correlation analysis. The ACK-M1 family constituted 21% of the Actinobacteria phylum, and members of this family are known to survive under nutrient-depleted conditions due to their efficient nutrient uptake (nitrogen, phosphorus and carbon) capabilities [64]. ASV_7 and ASV_18 were *Dolichospermum* sp. influenced by chlorophyll-a and TPP. This correlation confirmed that cyanobacterial blooms in Missisquoi Bay were associated with TPP, chlorophyll-a and pH. These environmental variables were, in turn, negatively correlated to NH_3_ and 1-day cumulative rain (Figure 9).

In 2018, the most influential variables on the bacterial community were NH_3_, TN, DOC (dissolved organic carbon), chlorophyll-a, pH, air temperature, water temperature and atmospheric pressure. NH_3_ and atmospheric pressure were correlated with the non-bloom community, whereas TN and chlorophyll-a had the strongest correlation with the blooms involving *Dolichospermum* species (ASVs 7 and 18). These environmental variables could explain 29.2% of the variation observed in the microbial community (RDA1: 19.3%, RDA2: 9.9%, adjusted R^2^ = 36.3%) (Figure 10).

### 3.6. Cyanobacterial Response to Environmental Variables

**Figure 11:**
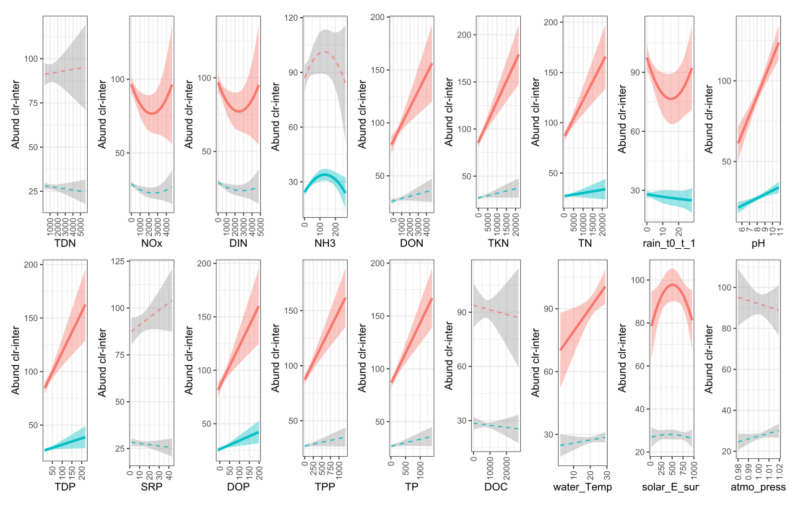
Response of *Dolichospermum* and *Microcystis* to environmental variables (red lines represent *Dolichospermum*; blue lines represent *Microcystis)*. Significant relationships are shown by solid lines and coloured confidence intervals.

We analyzed the relationship of the most dominant cyanobacterial genera, *Dolichospermum* and *Microcystis*, with environmental variables using a Latent Variable Model (LVM) framework (Figure 11).

*Dolichospermum* and *Microcystis* both had significant linear correlations with TN, TDP, DOP and pH. These two genera did not have a significant correlation with TDN, SRP, DOC or atmospheric pressure. *Dolichospermum* alone had a significant linear relationship with DON, TKN, TPP, TP and water temperature and a non-linear relationship with NOx, DIN, rain and solar irradiation. *Microcystis* had a significant linear relationship with 1-day cumulative rain and a non-linear relationship with NH_3_.

The correlation between *Dolichospermum* and DON, TKN and TPP could be explained by the extreme abundance of this genus in bloom samples. The organic and particulate N and P fractions during those bloom episodes were therefore associated with the cyanobacterial biomass rather than the presence of fresh bioavailable substrates ready for uptake by microorganisms.

## 4. Discussion

The influence of nutrients (N and P fractions), precipitation and temperature on the diversity and occurrence of CBs in Lake Champlain was investigated using thorough physicochemical analyses and high-throughput 16S rRNA gene amplicon sequencing and evaluated using statistical tools. Five locations in Missisquoi Bay, Lake Champlain, including a tributary river, the river mouth, pelagic, littoral and shoreline stations were monitored from April to November 2017 and 2018. The range of sampling sites allowed us to track the source and the fate of nutrients more effectively. The overall findings provided deeper insights into the influences of precipitation, agricultural and municipal practices on acute nutrient fluxes of N and P into the river and lake and on the impacts of bioavailable nutrients on the incidence of CBs.

### 4.1. Sources of Nutrients and Their Fate Influenced by Precipitation and Temperature

Manure and urea-based fertilizers are commonly used by farmers in the vicinity of our sampling sites. Urea-based fertilizers are rich in organic N and carbon, whereas manure and sewage are both rich in organic P, SRP, organic N and NH_3_ [9,10]. Fertilizing the soil in the spring or fall is a common practice to condition the soil for crop growth or prepare the land for the next spring. Manure application decisions are often triggered by the need to empty storage structures to reduce the risk of overflows. It is typically performed in the spring and in the fall. It can also be performed during the summer following forage harvest such as hay. In our study, the substantial peaks of nutrients observed in our water samples in April, specifically the organic N (DON and TKN) in PR, could be related to carry-over of nutrients from agricultural land via melting snow. The drastic inputs of nutrients identified in the river in June, August and October were likely associated to CSOs following intense precipitation or overflows associated with other tasks performed in the wastewater treatment plant such as emergencies or repair work. The nutrients observed in August and October could also have originated from runoffs (via surface and subsurface drainage systems) of manure applied on agricultural lands following the harvest of forage and crops.

We found a positive influence of rain on the soluble and inorganic fractions of N and P rather than the organic and particulate fractions. The Spearman correlation analysis showed that DOP was the only nutrient negatively correlated with rain, whereas the dissolved N fractions (NOx, DIN and TDN) were positively correlated. NOx and DIN were negatively correlated with air and water temperature indicating that their influxes were higher during the spring, as observed in the profile of nutrient concentrations. Strong correlations between chlorophyll-a, pH, TKN, TPP and TP were concomitant with the occurrence of intense CBs, where chlorophyll-a, TKN and TPP were associated specifically with cyanobacterial biomass. RDA analyses also confirmed this correlation between *Dolichospermum* sp. and TPP, chlorophyll-a and pH. 

While high N and P concentrations were measured in early spring samples, CBs started to occur in mid-summer as the water temperature reached 23–25 °C and worsened in August and September with increasing temperature. This observation is well supported by previous work reporting that high temperature (generally above 25 °C) is one of the most critical factors that accelerates cyanobacterial growth [63]. The spikes of dissolved nitrogen concentrations observed in the river in mid-June for both years after heavy precipitations (of between 49.3 and 100.1 mm of 7-day cumulative rain) contributed to the development of the first CB episode in the bay. Substantial input of nutrients in August and October 2017 corresponded to the period prior to intense bloom events indicating the significant contribution of bioavailable nutrients for the increase of cyanobacterial growth.

The mutual impacts of nutrient (N and P) concentrations and atmospheric variables such as mean annual temperature and precipitation as well as solar irradiance on CBs were previously demonstrated in controlled experiments and field samples [1,65,66]. Studies on nutrient–temperature dynamics showed that nutrients were the primary regulators of cyanobacterial growth and high temperatures accelerated their growth rate [67]. Toxic CBs were negatively correlated with rain (7-day cumulative rainfall of >50 mm) and positively correlated with temperatures above 20 °C [18]. Global warming in cold (Arctic and subarctic) climates contributes to early warming of water in the spring, and extended days of warmth in the fall are related to prolonged ice-free periods. Shallow eutrophic lakes are therefore subjected to a greater influence of high temperature and nutrients [63]. Reduction of water levels due to extreme heat contributes to the concentration of bioavailable nutrients [68]. During the 2017–2019 sampling seasons, the onset of CBs in Lake Champlain was recorded three weeks earlier than that of the previous year (30 August 2017, 8 August 2018 and 15 July 2019) and was related to early increases in air and water temperatures. We also experienced an important decrease in water levels towards the end of our sampling campaigns for those years where we could no longer access the shallowest sampling site (PRM) by boat. The concentration of bioavailable nutrients due to heat and the lysis of cyanobacteria from the first bloom event combined with decreasing water levels could be an explanation for the blooms of *Microcystis* that we now typically see in the fall. Low water levels would both enrich and ease the access to nutrients, specifically phosphorus from the sediments. 

Outputs of long-term monitoring data and water quality models predict intensifying rainfall in summer followed by extended periods of drought. While severe storms may lead to wash-off of the cyanobacteria at first glance, they also enhance agricultural runoff of nutrients and CSOs to the water bodies. Prolonged drought periods are anticipated to cause concentration of the nutrients that favor cyanobacterial growth [21,22,62,63]. This scenario matches with our observations where nutrient spikes were observed after cumulative precipitations of 48 to 100 mm and followed by CBs after a dry period (0 to 4 mm of 7-day cumulative rain).

### 4.2. How Bioavailable Nutrients Triggered Cyanobacterial Blooms

Nutrient concentrations were higher in the river samples (PR) and the river mouth (PRM) and more diluted in the bay samples (St1, St2, SBL), except for the episodes of intense bloom. PR is a major tributary to the bay thus, a major source of nutrients. Despite high nutrient concentrations in the river, cyanobacterial growth was not observed in these samples. An experiment performed in 2015 with floating buoys launched in PR corroborated the high flow rate in the river compared to the more stagnant conditions in the bay (Jean-Baptiste Burnet, personal communication). This experiment supported the hypothesis that nutrients originating from the river could rapidly (within a day) reach the bay specifically when the flow rate is high.

Seasonal NOx concentrations varied in PR in 2017 and 2018 samples. Peak levels of NOx in PR were observed in the spring, mid-June, mid-August and late October samples for both years following rain events. These results indicated that the organic load carried into the river could originate from the runoff (surface and subsurface drainage systems) of chemical fertilizers from municipal and agriculture activities. The impact of high nitrate concentrations under conditions promoting high algal growth was reported previously [69]. While this was not the scope of our study, algal dynamics would be worth investigating based on the high NOx concentrations in our spring samples.

PR and PRM nutrient profiles were similar in 2017 and 2018, except for the bloom samples. NH_3_ levels were higher in the bay (PRM, St1, St2) in June and October 2017, as well as in June and mid-August 2018. The June concentrations could likely be attributed to CSOs for both years since concentrations above 15 mg N/l and charges greater than 38 kg/d were recorded for the month [19]. Increases in NH_3_ in the October 2017 and mid-August 2018 samples corresponded to the collapse of the first cyanobacterial bloom episodes. The affinity of *Microcystis* towards NH_3_ was reviewed by Gobler et al. [70]. The second bloom of *Microcystis* in November 2017 could have emerged from the high recycling rate of reduced N by *Microcystis*, in this case, the ammonium released after the *Dolichospermum* bloom die-off [70].

In our study, TKN and TPP had substantial influence on overall TN and TP concentrations, respectively, and were strongly associated with cyanobacterial biomass; therefore, the highest concentrations were recorded during bloom events. High SRP levels during bloom periods indicated a continuous bioavailability of P.

We investigated the ratios between various N and P fractions (Table S4) but could not establish a specific TN:TP ratio that could predict the occurrence of CBs in Missisquoi Bay primarily because of the influence of cyanobacterial cells on organic and particulate forms of N and P and, thus, on TN and TP concentrations. While cyanobacterial dominance is typically expected under N-limited conditions [61], only 5 out of 41 bloom samples in our study had TN:TP ratio between 2 and 20 (N-limited). The lowest TN:TP ratio of 2 was recorded in 2017 due to extreme TP and TPP concentrations as a result of a manure or septic waste spill. This spill could not be associated with an intense rainfall event and was concomitant with an intense cyanobacterial bloom, high concentrations of *E. coli* and a massive mussel die-off. In a previous study performed in 2009 in Missisquoi Bay, we established that TN:TP ratios approaching 11:1 coupled with an increase in temperature promoted the growth of toxin-producing *Microcystis* [2]. 

Similarly, we did not find a correlation between CBs and DIN:SRP ratios where DIN:SRP < 10:1 were considered to indicate strongly nitrogen-limiting conditions that favoured the growth and proliferation of N_2_-fixing cyanobacteria [71]. In our study, only nine bloom samples had DIN:SRP ratio below 10.

### 4.3. Nutrient Preferences of Cyanobacteria

Niche separation between *Dolichospermum* and *Microcystis* in Lake Champlain, Missisquoi Bay, was previously investigated based on the total and dissolved fractions of N and P [34]. Different dynamics of *Dolichospermum* and *Microcystis* at the strain level, depending on nutrient fractions and precipitation, were reported.

In this study, the impact of various forms of nutrients and environmental parameters on *Dolichospermum* and *Microcystis*, two major genera identified in the bay during the 2017 and 2018 bloom events, were analyzed. We observed that the influence of nutrient–temperature–precipitation was more predominant during the first intense bloom of the season (30 August 2017 and 8 August 2018) compared to the subsequent blooms. The self-perpetuating nature of the system could possibly be explained by intermittent fluxes of external nutrients, upwelling from sediments, endogenous decay as well as cellular N and P storage mechanisms of cyanobacteria [72].

*Dolichospermum* and *Microcystis* showed a significant linear correlation with TN, TDP, DOP and pH. We therefore can imply that dissolved P (organic and inorganic) was the major P source for their growth. Increases in pH were observed during cyanobacterial growth most likely due to the consumption of inorganic carbon [73]. The significant correlations between *Dolichospermum* and DON, TKN, TPP and TP concentrations were most likely due to bloom episodes when organic and particulate nutrients measured in the water column originated from the cyanobacterial biomass.

Similar to other work, high water temperature was significant for the growth of *Dolichospermum* in the bay [18]. Considering the energy demand for metabolism, cyanobacteria prefer to use the most reduced form of N, which is ammonium. The relationship established between *Microcystis* and NH_3_ by other studies supports our observations that the *Microcystis* blooms took place in the presence of high concentrations of NH_3_ in the water column [70]. In this study, the impact of NH_3_ on *Microcystis* dominance during the second bloom events was observed following the manure or septic waste spill at the end of the summer 2017 as well as the lysis of the *Dolichospermum* blooms.

Based on previous research [30], it is very unlikely that *Dolichospermum* would take advantage of its N_2_ fixation mechanism because it is a fairly energy intensive process requiring 16 moles of adenosine triphosphate to reduce each mole of nitrogen [74].

Studies performed in the Missisquoi Bay area for Lake Champlain reported very low N_2_ fixation rates in areas very close to our sampling stations [30]. They found an ammonium regeneration rate of 9.8 t N/day, which was almost twice the N input from the tributaries. Similar observations were reported by other studies [75]. This regeneration mechanism could explain high cyanobacterial growth rates despite low ammonium concentrations in the samples that we collected in early summer [30,72,75].

Despite the previous hypothesis that P was the limiting nutrient of CBs, the dual impact of N and P has been well explained in the literature [76,77,78,79]. Studies showed that *Dolichospermum* are more dominant in low N, high P, whereas *Microcystis* grows well under high N, low P conditions [70,80]. Mutual dependence of *Dolichospermum* on N and P has been described, where excess P promoted N_2_ fixation and excess N favoured alkaline phosphatase activity to supply P from organic P [78]. We believe that strategies based on controlling single nutrients could enhance the growth of other cyanobacteria especially in waterbodies such as Missisquoi Bay that harbour such an impressive species diversity. For example, taxonomic identification by microscopy performed in 2017 and 2018 revealed 42 and 36 cyanobacterial species, respectively.

### 4.4. Dynamics between Cyanobacterial and Heterotrophic Bacterial Communities

It is clear that the microbial community in Missisquoi Bay undergoes complex dynamics and multiple transformations throughout the year. The bacteria have the ability to outcompete each other mainly based on the carbon source, nutrient composition, temperature and the capacity to conduct photosynthesis. Heterotrophic bacteria such as *Candidatus Xiphinematobacter, Flavobacterium, Fluviicola, Limnohabitans, Polynucleobacter* and *Sediminibacterium* were present during bloom as well as non-bloom periods. Interestingly, they all have the ability to use various forms of nutrients, specifically urea, nitrate and ammonia [64,81,82,83]. Previous research showed their capabilities to grow on algal polysaccharides [84] and to degrade cyanotoxins and cell debris [84,85]. The abundance of *Polynucleobacter* in pre-bloom samples and its potential as a bloom biomarker was previously reported for this sampling site [33]. *Limnohabitans* sp. (Betaproteobacteria) and *Cytophaga-Flavobacteria* (Bacteroidetes) were reported to occur in spring algal blooms [86]. Both taxa grow on algal exudates [84]. *Flavobacterium* and *Fluviicola* were abundant in bloom and non-bloom samples. *Flavobacterium* could potentially be involved in metabolizing cell debris, lysing *Microcystis* cells and degrading cyanotoxins [84,85]. *Candidatus Xiphinematobacter* (Verrucomicrobia) possesses various metabolic capabilities in especially psychrophilic conditions, such as degrading chlorophyll related products, urea [87] and algal polysaccharides [84]. *Polaromonas* sp. was previously reported in glacial environments, where its abundance increased in surface waters due to melting ice and snow in the spring [88]. Similarly, the high abundance of *Rhodoferax* in both spring and fall, and low abundance in the summer, was found in an ice-covered river around Quebec City, Canada [88], which experiences a similar climate to that of our sampling site.

The presence of such a high diversity of bacteria in Missisquoi Bay with various potentials related to nitrogen metabolism, degradation of algal exudates, cell debris and cyanotoxins indicate mutual benefits between the cyanobacterial and non-cyanobacterial communities and is worthy of further study. The abundance of bacteria involved in the metabolism of nitrogenous compounds compared to that of phosphorus indicates the significance of nitrogen on overall microbial dynamics as well as CB formation in the bay.

## 5. Conclusions

Agricultural runoff and CSOs contribute to sustaining the poor water quality of Missisquoi Bay. These events are enhanced during periods of heavy precipitation. Bioavailable fractions of both nitrogen and phosphorus have a critical impact on the occurrence of CBs due to their high regeneration rates. In addition, POPs including pesticides and herbicides originating from agricultural practices and municipal activities as well as wastewater can also trigger blooms. Corrective measures in the sewage network and wastewater treatment facilities should therefore be funded and implemented rapidly to eliminate CSOs and the corresponding release of nutrients, POPs, *E. coli* and pathogens in aquatic ecosystems as well as in lakes and reservoirs that are a source of drinking water. Similarly, application of chemical fertilizers and manure onto agricultural fields can enhance the transfer of nutrients to the river and carryover into the lake. The relationship between nutrients, precipitation or drought periods and CBs was verified in this study. Substantial peaks of nutrients following intense precipitation confirmed the importance of employing and implementing measures to reduce soil erosion, surface and subsurface runoff, including same day incorporation or injection of manure especially when heavy or extended precipitation is in the forecast.

The diversity of cyanobacterial species in Missisquoi Bay is impressive and inevitably concomitant with the ability to use a variety of bioavailable sources of both N and P. We therefore believe that corrective measures to reduce both N and P loads in the bay should be implemented to help control and/or eliminate cyanobacterial blooms.

## Figures and Tables

**Table 1:** Total number of samples taken in 2017 and 2018.

Site Name	Description	Total Number of Samples	
		2017	2018
PR	Pike River	15	13
PRM	Pike River Mouth	10	8
St1	Littoral Station	14	12
St2	Pelagic Station	10	8
SBL	Shore Boat Launch	8	6

**Table 2:** Spatio-temporal analysis using Permanova. The best explanatory variables for microbial diversity are presented in italics.

	Permanova		Dispersion
	R^2^	*p*	
Depth	0.109	0.001	0.364
Site	0.167	0.001	0.006
Day	*0.424*	*0.001*	*0.946*
Week	*0.425*	*0.001*	*0.859*
Month	0.215	0.001	0.321
Season	0.105	0.001	0.356
Years	0.017	0.059	0.101

## Data Availability

The raw sequence files were submitted to NCBI with the BioProject number PRJNA744879 and Sequence Read Archive (SRA) number SUB9973256.

## Supplementary Materials

The following are available online at https://www.mdpi.com/article/10.3390/microorganisms9102097/s1, Figure S1: Correlation analysis of environmental variables—2017; Figure S2: Correlation analysis of environmental variables—2018; Figure S3: Distribution of cyanobacteria per sampling site; Figure S4: Abundance of genera per sampling site; Figure S5: Alpha diversity richness breakaway; Table S1: Environmental variables and bloom status; Table S2: Concentration ranges of nitrogen fractions in 2017 and 2018; Table S3: Concentration ranges of phosphorus fractions in 2017 and 2018; Table S4: TN:TP and DIN:SRP ratios in bloom and non-bloom conditions; Table S5: Precipitation profile during the sampling period; Table S6: RDA analyses of significant environmental variables and bacterial community for 2017 and 2018.

## References

[1] Jankowiak J., Hattenrath-Lehmann T., Kramer B.J., Ladds M., Gobler C.J. (2019). Deciphering the effects of nitrogen, phosphorus, and temperature on cyanobacterial bloom intensification, diversity, and toxicity in western Lake Erie. Limnol. Oceanogr..

[2] Fortin N., Munoz-Ramos V., Bird D., Levesque B., Whyte L.G., Greer C.W. (2015). Toxic cyanobacterial bloom triggers in missisquoi bay, lake champlain, as determined by next-generation sequencing and quantitative PCR. Life.

[3] Marmen S., Blank L., Al-Ashhab A., Malik A., Ganzert L., Lalzar M., Grossart H.P., Sher D. (2020). The Role of Land Use Types and Water Chemical Properties in Structuring the Microbiomes of a Connected Lake System. Front. Microbiol..

[4] Facey D.E., Marsden J.E., Mihuc T.B., Howe E.A. (2012). Lake Champlain 2010: A summary of recent research and monitoring initiatives. J. Great Lakes Res..

[5] Smeltzer E., Shambaugh A.D., Stangel P. (2012). Environmental change in Lake Champlain revealed by long-term monitoring. J. Great Lakes Res..

[6] Bakhsh A., Kanwar R.S., Bailey T.B., Cambardella C.A., Karlen D.L., Colvin T.S. (2002). Cropping System Effects on NO_3_–N Loss with Subsurface Drainage Water. Trans. ASAE.

[7] Michaud A.R., Poirier S.-C., Whalen J.K. (2019). Tile Drainage as a Hydrologic Pathway for Phosphorus Export from an Agricultural Subwatershed. J. Environ. Qual..

[8] Michalak A.M., Anderson E.J., Beletsky D., Boland S., Bosch N.S., Bridgeman T.B., Chaffin J.D., Cho K., Confesor R., Daloglu I. (2013). Record-setting algal bloom in Lake Erie caused by agricultural and meteorological trends consistent with expected future conditions. Proc. Natl. Acad Sci. USA.

[9] Munroe J. (2018). Soil Fertility Handbook Publication 611.

[10] Mudge S.M., Ball A.S., Morrison R.D., Murphy B.L. (2006). Sewage. Environmental Forensics: Contaminant Specific Guide.

[11] Harris T.D., Smith V.H. (2016). Do persistent organic pollutants stimulate cyanobacterial blooms?. Inland Waters.

[12] Liu Y., Gao B., Yue Q., Guan Y., Wang Y., Huang L. (2012). Influences of two antibiotic contaminants on the production, release and toxicity of microcystins. Ecotoxicol. Environ. Saf..

[13] Stoichev T., Baptista M.S., Basto M.C.P., Vasconcelos V.M., Vasconcelos M.T.S.D. (2011). Effects of minocycline and its degradation products on the growth of Microcystis aeruginosa. Ecotoxicol. Environ. Saf..

[14] Brezovšek P., Eleršek T., Filipič M. (2014). Toxicities of four anti-neoplastic drugs and their binary mixtures tested on the green alga Pseudokirchneriella subcapitata and the cyanobacterium Synechococcus leopoliensis. Water Res..

[15] Proia L., Osorio V., Soley S., Köck-Schulmeyer M., Pérez S., Barceló D., Romaní A.M., Sabater S. (2013). Effects of pesticides and pharmaceuticals on biofilms in a highly impacted river. Environ. Pollut..

[16] Liu Y., Chen X., Zhang J., Gao B. (2015). Hormesis Effects of Amoxicillin on Growth and Cellular Biosynthesis of Microcystis aeruginosa at Different Nitrogen Levels. Microb. Ecol..

[17] Michaud A.R., Niang M.A. (2019). Analyse Coûts-Efficacité des Actions Proposées Pour Réduire de 40% les Charges de Phosphore de la Rivière la Roche à la Baie Missisquoi.

[18] Haakonsson S., Rodriguez-Gallego L., Somma A., Bonilla S. (2017). Temperature and precipitation shape the distribution of harmful cyanobacteria in subtropical lotic and lentic ecosystems. Sci. Total. Environ..

[19] Ministère de l’Environnement et de la Lutte Contre les Changements Climatiques. https://www.environnement.gouv.qc.ca/..

[20] Bartosiewicz M., Przytulska A., Deshpande B.N., Antoniades D., Cortes A., MacIntyre S., Lehmann M.F., Laurion I. (2019). Effects of climate change and episodic heat events on cyanobacteria in a eutrophic polymictic lake. Sci. Total Environ..

[21] Paerl H.W., Paul V.J. (2012). Climate change: Links to global expansion of harmful cyanobacteria. Water Res..

[22] Reichwaldt E.S., Ghadouani A. (2012). Effects of rainfall patterns on toxic cyanobacterial blooms in a changing climate: Between simplistic scenarios and complex dynamics. Water Res..

[23] Coffey R., Paul M., Stamp J., Hamilton A., Johnson T. (2018). A Review of Water Quality Responses to Air Temperature and Precipitation Changes 2: Nutrients, Algal Blooms, Sediment, Pathogens. J. Am. Water Resour. Assoc..

[24] Ahn C.-Y., Chung A.-S., Oh H.-M. (2002). Rainfall, phycocyanin, and N:P ratios related to cyanobacterial blooms in a Korean large reservoir. Hydrobiologia.

[25] Hendry K., Sambrook H., Underwood C., Waterfall R., Williams A. (2006). Eutrophication of Tamar Lakes (1975–2003): A case study of land-use impacts, potential solutions and fundamental issues for the Water Framework Directive. Water Environ. J..

[26] Burford M.A., Johnson S.A., Cook A.J., Packer T.V., Taylor B.M., Townsley E.R. (2007). Correlations between watershed and reservoir characteristics, and algal blooms in subtropical reservoirs. Water Res..

[27] Mrdjen I., Fennessy S., Schaal A., Dennis R., Slonczewski J.L., Lee S., Lee J. (2018). Tile Drainage and Anthropogenic Land Use Contribute to Harmful Algal Blooms and Microbiota Shifts in Inland Water Bodies. Environ. Sci. Technol..

[28] Michaud A.R., Laverdière M.R. (2004). Cropping, soil type and manure application effects on phosphorus export and bioavailability. Can. J. Soil Sci..

[29] Poirier S.-C., Whalen J.K., Michaud A.R. (2012). Bioavailable Phosphorus in Fine-Sized Sediments Transported from Agricultural Fields. Soil Sci. Soc. Am. J..

[30] McCarthy M.J., Gardner W.S., Lehmann M.F., Bird D.F. (2013). Implications of water column ammonium uptake and regeneration for the nitrogen budget in temperate, eutrophic Missisquoi Bay, Lake Champlain (Canada/USA). Hydrobiologia.

[31] McCarthy M.J., Gardner W.S., Lehmann M.F., Guindon A., Bird D.F. (2016). Benthic nitrogen regeneration, fixation, and denitrification in a temperate, eutrophic lake: Effects on the nitrogen budget and cyanobacteria blooms. Limnol. Oceanogr..

[32] Isles P.D.F., Giles C.D., Gearhart T.A., Xu Y., Druschel G.K., Schroth A.W. (2015). Dynamic internal drivers of a historically severe cyanobacteria bloom in Lake Champlain revealed through comprehensive monitoring. J. Great Lakes Res..

[33] Tromas N., Fortin N., Bedrani L., Terrat Y., Cardoso P., Bird D., Greer C.W., Shapiro B.J. (2017). Characterising and predicting cyanobacterial blooms in an 8-year amplicon sequencing time course. ISME J..

[34] Tromas N., Taranu Z.E., Martin B.D., Willis A., Fortin N., Greer C.W., Shapiro B.J. (2018). Niche Separation Increases with Genetic Distance among Bloom-Forming Cyanobacteria. Front. Microbiol..

[35] Google Earth. earth.google.com.

[36] Farm Zone. http://www.farmzone.com/.

[37] The Weather Network. https://www.theweathernetwork.com/ca.

[38] APHA (2005). Standard Methods For the Examination of Water and Wastewater.

[39] APHA (2012). Standard Methods for the Examination of Water and Wastewater.

[40] Joosten A.M.T. (2006). Flora of the Bluegreen Algae of the Netherlands: The Non-Filamentous Species of Inland Waters.

[41] Komárek J., Anagnostidis K. (1999). Cyanoprokaryota, Teil 1 Chroococcales.

[42] Komárek J. (2013). Cyanoprokaryota, 3rd Part, Heterocystous Genera.

[43] Hillebrand H., Dürselen C.-D., Kirschtel D., Pollingher U., Zohary T. (1999). Biovolume Calculation for Pelagic and Benthic Microalgae. J. Phycol..

[44] Preheim S.P., Perrotta A.R., Martin-Platero A.M., Gupta A., Alm E.J. (2013). Distribution-based clustering: Using ecology to refine the operational taxonomic unit. Appl. Environ. Microbiol..

[45] Callahan B.J., McMurdie P.J., Rosen M.J., Han A.W., Johnson A.J.A., Holmes S.P. (2016). DADA2: High-resolution sample inference from Illumina amplicon data. Nat. Methods.

[46] McMurdie P.J., Holmes S. (2013). Phyloseq: An R Package for Reproducible Interactive Analysis and Graphics of Microbiome Census Data. PLoS ONE.

[47] Newton R.J., Jones S.E., Eiler A., McMahon K.D., Bertilsson S. (2011). A guide to the natural history of freshwater lake bacteria. Microbiol. Mol. Biol. Rev..

[48] Rohwer R.R., Hamilton J.J., Newton R.J., McMahon K.D. (2018). TaxAss: Leveraging a Custom Freshwater Database Achieves Fine-Scale Taxonomic Resolution. Msphere.

[49] Willis A.D., Martin B.D. (2020). Estimating diversity in networked ecological communities. Biostatistics.

[50] Willis A., Bunge J. (2015). Estimating diversity via frequency ratios. Biometrics.

[51] Willis A., Bunge J., Whitman T. (2017). Improved detection of changes in species richness in high diversity microbial communities. J. R. Stat. Soc. Ser. C (Appl. Stat.).

[52] Fuglede B., Topsoe F. Jensen-Shannon Divergence and Hilbert Space Embedding. Proceedings of the International Symposium on Information Theory (ISIT).

[53] Anderson M.J. (2001). A new method for non-parametric multivariate analysis of variance. Austral. Ecol..

[54] Anderson M.J. (2006). Distance-Based Tests for Homogeneity of Multivariate Dispersions. Biometrics.

[55] Oksanen J., Blanchet F., Kindt R., Legendre P., Minchin P., O’Hara R., Simpson G., Solymos P., Henry M., Stevens H. (2016). Vegan: Community Ecology Package. R Package.

[56] Rao C.R. (1964). The Use and Interpretation of Principal Component Analysis in Applied Research. Sankhyā Indian J. Stat. Ser. A.

[57] Rao C.R. (1995). A review of canonical coordinates and an alternative to correspondence analysis using Hellinger distance. Qüestiió.

[58] Legendre P., Legendre L. (1998). Numerical Ecology.

[59] Aitchison J. (1982). The Statistical Analysis of Compositional Data. J. R. Stat. Soc. Ser. B (Methodol.).

[60] Paliy O., Shankar V. (2016). Application of multivariate statistical techniques in microbial ecology. Mol. Ecol..

[61] Hecky R.E., Campbell P., Hendzel L.L. (1993). The stoichiometry of carbon, nitrogen, and phosphorus in particulate matter of lakes and oceans. Limnol. Oceanogr..

[62] Chapra S.C., Boehlert B., Fant C., Bierman V.J., Henderson J., Mills D., Mas D.M.L., Rennels L., Jantarasami L., Martinich J. (2017). Climate Change Impacts on Harmful Algal Blooms in U.S. Freshwaters: A Screening-Level Assessment. Environ. Sci. Technol..

[63] Huisman J., Codd G.A., Paerl H.W., Ibelings B.W., Verspagen J.M.H., Visser P.M. (2018). Cyanobacterial blooms. Nat. Rev. Microbiol..

[64] Ghai R., Mizuno C.M., Picazo A., Camacho A., Rodriguez-Valera F. (2014). Key roles for freshwater Actinobacteria revealed by deep metagenomic sequencing. Mol. Ecol..

[65] Chen M., Zeng G., Zhang J., Xu P., Chen A., Lu L. (2015). Global Landscape of Total Organic Carbon, Nitrogen and Phosphorus in Lake Water. Sci. Rep..

[66] Zhou Q., Zhang Y., Lin D., Shan K., Luo Y., Zhao L., Tan Z., Song L. (2016). The relationships of meteorological factors and nutrient levels with phytoplankton biomass in a shallow eutrophic lake dominated by cyanobacteria, Lake Dianchi from 1991 to 2013. Environ. Sci. Pollut. Res. Int..

[67] Rigosi A., Carey C.C., Ibelings B.W., Brookes J.D. (2014). The interaction between climate warming and eutrophication to promote cyanobacteria is dependent on trophic state and varies among taxa. Limnol. Oceanogr..

[68] Paerl H.W., Havens K.E., Hall N.S., Otten T.G., Zhu M., Xu H., Zhu G., Qin B. (2020). Mitigating a global expansion of toxic cyanobacterial blooms: Confounding effects and challenges posed by climate change. Mar. Freshw. Res..

[69] Donald D.B., Bogard M.J., Finlay K., Leavitt P.R. (2011). Comparative effects of urea, ammonium, and nitrate on phytoplankton abundance, community composition, and toxicity in hypereutrophic freshwaters. Limnol. Oceanogr..

[70] Gobler C.J., Burkholder J.M., Davis T.W., Harke M.J., Johengen T., Stow C.A., Van de Waal D.B. (2016). The dual role of nitrogen supply in controlling the growth and toxicity of cyanobacterial blooms. Harmful Algae.

[71] Havens K.E., James R.T., East T.L., Smith V.H. (2003). N:P ratios, light limitation, and cyanobacterial dominance in a subtropical lake impacted by non-point source nutrient pollution. Environ. Pollut..

[72] Hampel J.J., McCarthy M.J., Neudeck M., Bullerjahn G.S., McKay R.M.L., Newell S.E. (2019). Ammonium recycling supports toxic Planktothrix blooms in Sandusky Bay, Lake Erie: Evidence from stable isotope and metatranscriptome data. Harmful Algae.

[73] Li J., Hansson L.-A., Persson K. (2018). Nutrient Control to Prevent the Occurrence of Cyanobacterial Blooms in a Eutrophic Lake in Southern Sweden, Used for Drinking Water Supply. Water.

[74] Wagner S.C. (2011). Biological Nitrogen Fixation. Nat. Educ. Knowl..

[75] Hampel J.J., McCarthy M.J., Gardner W.S., Zhang L., Xu H., Zhu G., Newell S.E. (2018). Nitrification and ammonium dynamics in Taihu Lake, China: Seasonal competition for ammonium between nitrifiers and cyanobacteria. Biogeosciences.

[76] Harke M.J., Davis T.W., Watson S.B., Gobler C.J. (2016). Nutrient-Controlled Niche Differentiation of Western Lake Erie Cyanobacterial Populations Revealed via Metatranscriptomic Surveys. Environ. Sci. Technol..

[77] Paerl H.W., Havens K.E., Xu H., Zhu G., McCarthy M.J., Newell S.E., Scott J.T., Hall N.S., Otten T.G., Qin B. (2019). Mitigating eutrophication and toxic cyanobacterial blooms in large lakes: The evolution of a dual nutrient (N and P) reduction paradigm. Hydrobiologia.

[78] Wang S., Xiao J., Wan L., Zhou Z., Wang Z., Song C., Zhou Y., Cao X. (2018). Mutual Dependence of Nitrogen and Phosphorus as Key Nutrient Elements: One Facilitates Dolichospermum flos-aquae to Overcome the Limitations of the Other. Environ. Sci. Technol..

[79] Levy S. (2017). Microcystis Rising: Why Phosphorus Reduction Isn’t Enough to Stop CyanoHABs. Environ. Health Perspect..

[80] Chia M.A., Jankowiak J.G., Kramer B.J., Goleski J.A., Huang I.S., Zimba P.V., do Carmo Bittencourt-Oliveira M., Gobler C.J. (2018). Succession and toxicity of Microcystis and Anabaena (Dolichospermum) blooms are controlled by nutrient-dependent allelopathic interactions. Harmful Algae.

[81] Chun S.J., Cui Y., Lee C.S., Cho A.R., Baek K., Choi A., Ko S.R., Lee H.G., Hwang S., Oh H.M. (2019). Characterization of Distinct CyanoHABs-Related Modules in Microbial Recurrent Association Network. Front. Microbiol..

[82] Kalyuzhnaya M.G., Beck D.A.C., Vorobev A., Smalley N., Kunkel D.D., Lidstrom M.E., Chistoserdova L. (2012). Novel methylotrophic isolates from lake sediment, description of Methylotenera versatilis sp. nov. and emended description of the genus Methylotenera. Int. J. Syst. Evol. Microbiol..

[83] Bosch G., Wang T., Latypova E., Kalyuzhnaya M.G., Hackett M., Chistoserdova L. (2009). Insights into the physiology of Methylotenera mobilis as revealed by metagenome-based shotgun proteomic analysis. Microbiology.

[84] Parulekar N.N., Kolekar P., Jenkins A., Kleiven S., Utkilen H., Johansen A., Sawant S., Kulkarni-Kale U., Kale M., Saebo M. (2017). Characterization of bacterial community associated with phytoplankton bloom in a eutrophic lake in South Norway using 16S rRNA gene amplicon sequence analysis. PLoS ONE.

[85] Cai H.Y., Yan Z.S., Wang A.J., Krumholz L.R., Jiang H.L. (2013). Analysis of the attached microbial community on mucilaginous cyanobacterial aggregates in the eutrophic Lake Taihu reveals the importance of Planctomycetes. Microb. Ecol..

[86] Salcher M.M., Pernthaler J., Frater N., Posch T. (2011). Vertical and longitudinal distribution patterns of different bacterioplankton populations in a canyon-shaped, deep prealpine lake. Limnol. Oceanogr..

[87] Tran P., Ramachandran A., Khawasik O., Beisner B.E., Rautio M., Huot Y., Walsh D.A. (2018). Microbial life under ice: Metagenome diversity and in situ activity of Verrucomicrobia in seasonally ice-covered Lakes. Environ. Microbiol..

[88] Cruaud P., Vigneron A., Fradette M.S., Dorea C.C., Culley A.I., Rodriguez M.J., Charette S.J. (2020). Annual bacterial community cycle in a seasonally ice-covered river reflects environmental and climatic conditions. Limnol. Oceanogr..

