# Supplementary material for: Bioavailable Nutrients (N and P) and Precipitation Patterns Drive Cyanobacterial Blooms in Missisquoi Bay, Lake Champlain
Source: Microorganisms. 2021 Oct 4;9(10):2097. doi: 10.3390/microorganisms9102097 (PMC8537112; doi:10.3390/microorganisms9102097)
Supplement: Supplementary file 1 [file microorganisms-09-02097-s001.zip › microorganisms-1411955-supplementary/Supplementary Figures-20210930.pdf]

Bioavailable nutrients (N and P) and precipitation patterns drive cyanobacterial blooms in Missisquoi Bay, Lake Champlain

Sukriye Celikkol[1*], Nathalie Fortin[2a], Nicolas Tromas[3a], Herinandrianina Andriananjamanantsoa[3], Charles W. Greer[1,2*]

Affiliations:

[1] Department of Natural Resource Sciences, McGill University, Sainte-Anne-de-Bellevue, QC, H9X 3V9 Canada

[2] Energy, Mining and Environment Research Centre, National Research Council Canada, Montreal, QC H4P 2R2 Canada

[3] Department of Biological Sciences, University of Montreal, Montreal, QC H2V 0B3 Canada

[a]These authors contributed equally to this work.

*Corresponding authors:

Sukriye Celikkol: celikkolsu@gmail.com

Charles W. Greer: Charles.Greer@nrc-cnrc.gc.ca

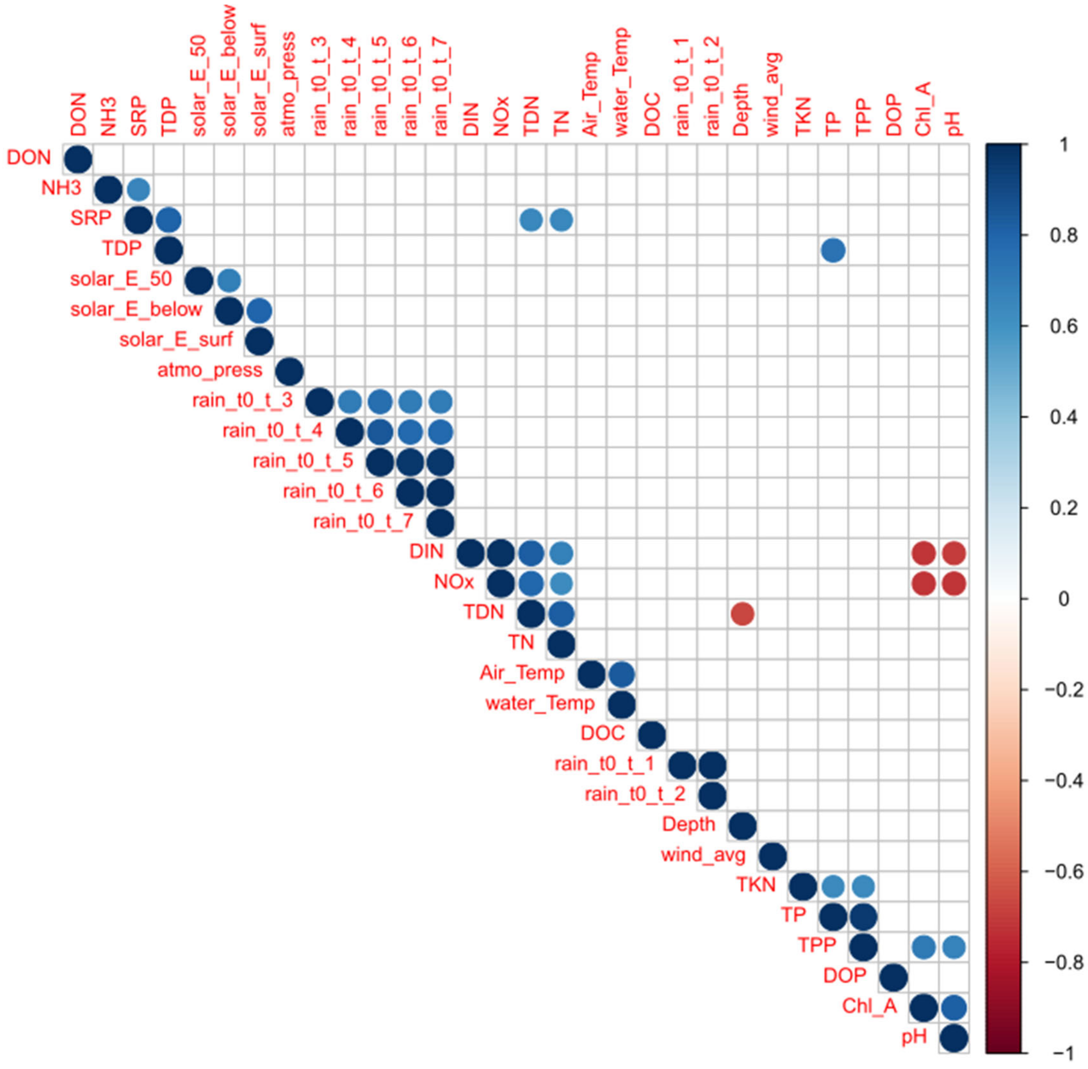

Figure S1. Correlation analysis of environmental variables -2017. The color scale indicates the strength of correlation between environmental parameters. Blue scale indicates positive correlations, red scale indicates negative correlations.

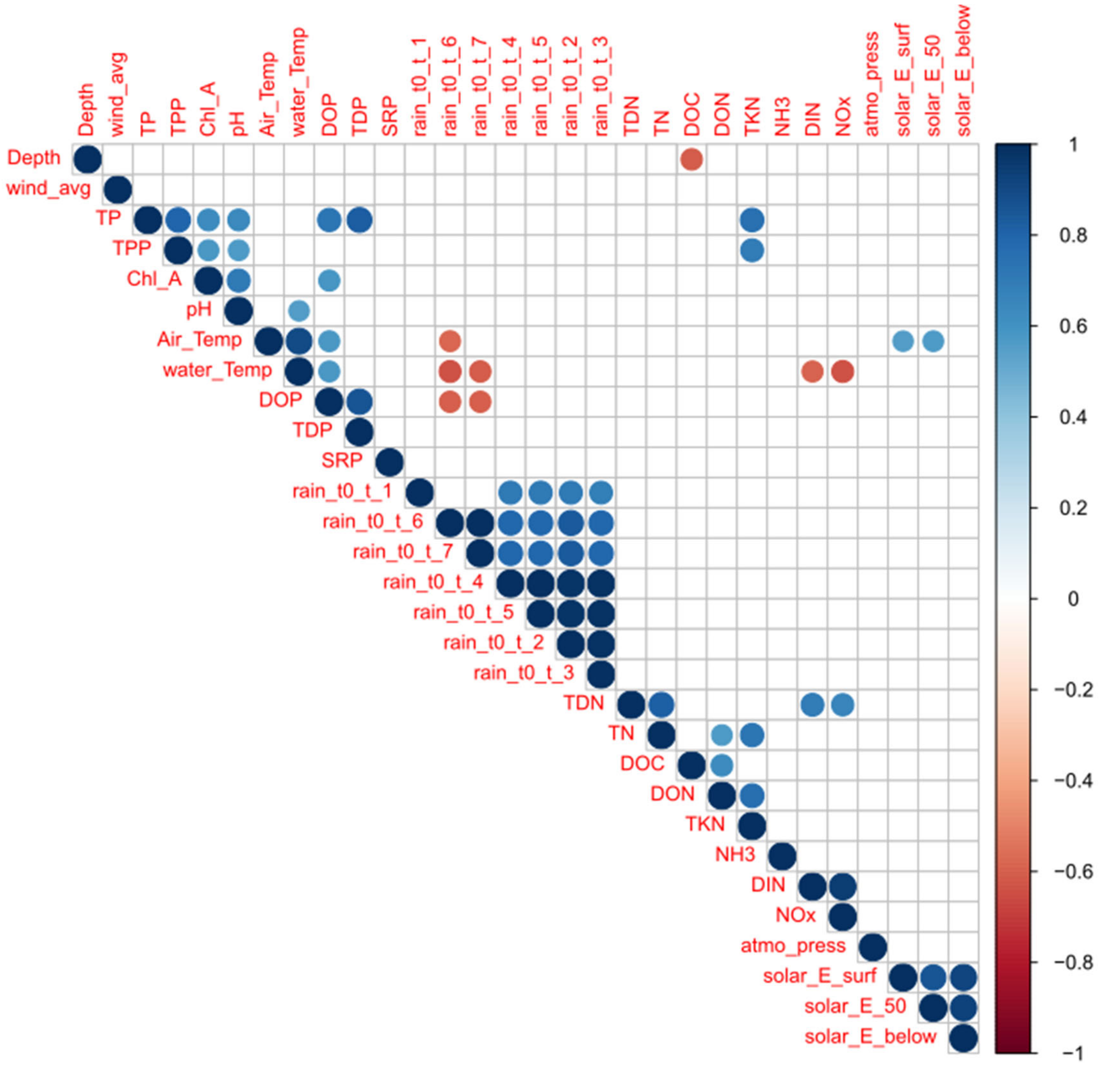

Figure S2. Correlation analysis of environmental variables -2018. The color scale indicates the strength of correlation between environmental parameters. Blue scale indicates positive correlations, red scale indicates negative correlations.

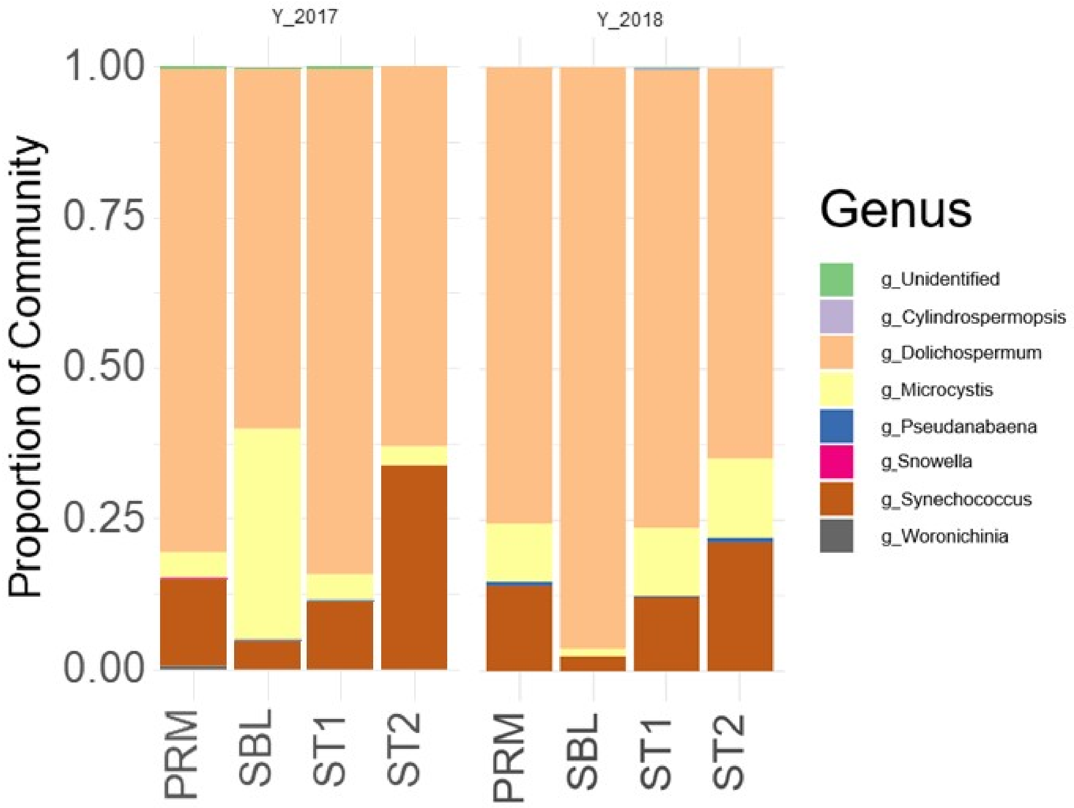

Figure S3. Distribution of cyanobacteria per sampling site. The cyanobacteria could be identified down to the genus level by 16S rRNA gene amplicon sequencing. The most dominant *Dolichospermum* and *Microcystis* species were identified by microscopy as *Dolichospermum flosaquae, D. crassum, D. spiroides, D. circinale, D. planctonicum, Microcystis sp, M. aeruginosa* and *M. wesenbergii.*

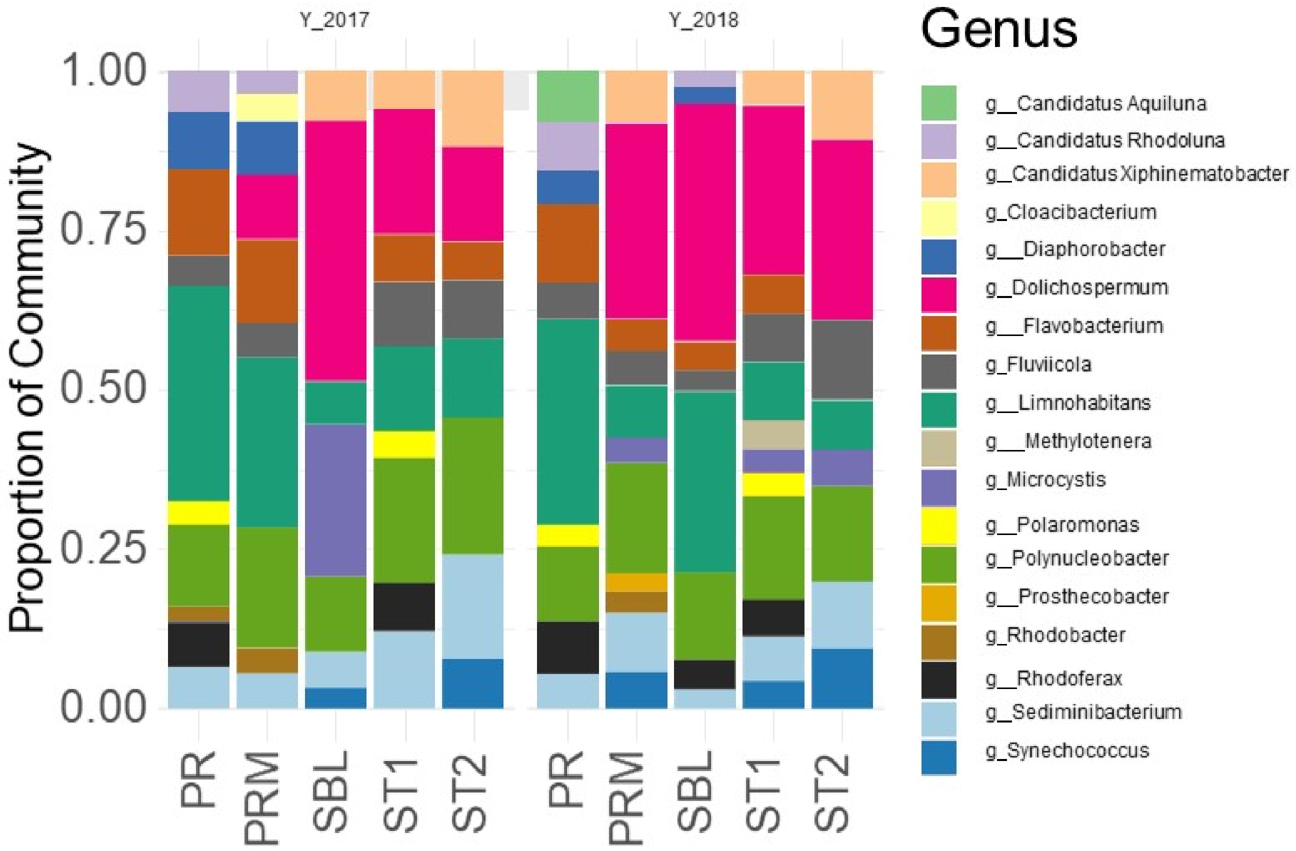

Figure S4. Abundance of genera per sampling site.

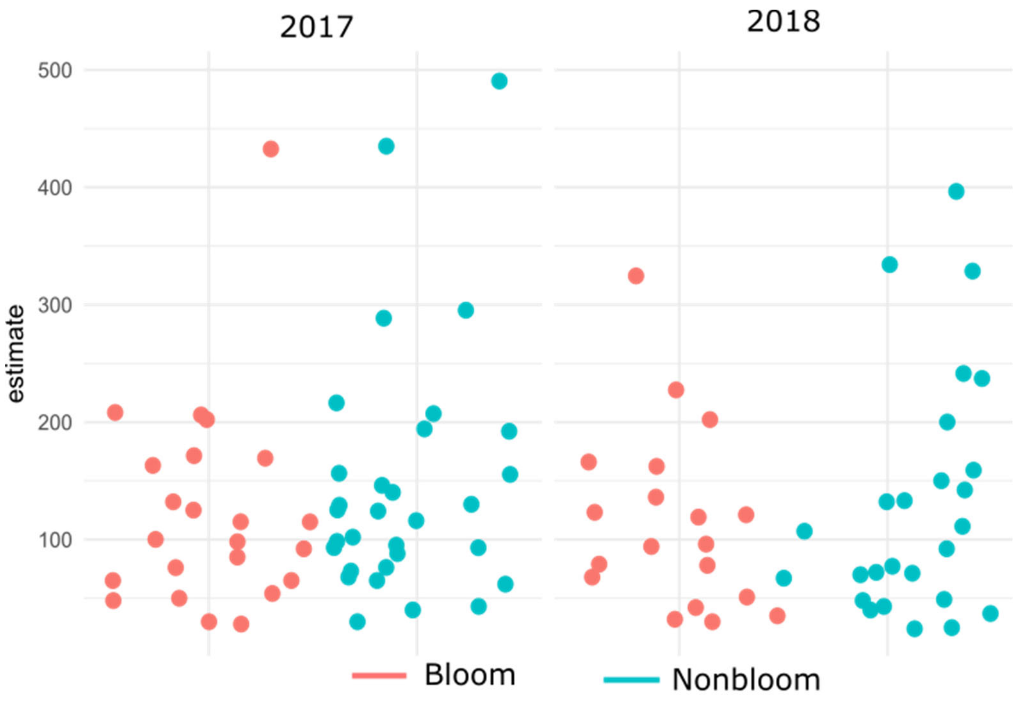

Figure S5. Alpha diversity richness breakaway. The species richness was similar during bloom and non-bloom periods.
